# Blood transcriptomics to characterize key biological pathways and identify biomarkers for predicting mortality in melioidosis

**DOI:** 10.1080/22221751.2020.1858176

**Published:** 2021-01-17

**Authors:** Thatcha Yimthin, Jacqueline Margaret Cliff, Rungnapa Phunpang, Peeraya Ekchariyawat, Taniya Kaewarpai, Ji-Sook Lee, Clare Eckold, Megan Andrada, Ekkachai Thiansukhon, Kittisak Tanwisaid, Somchai Chuananont, Chumpol Morakot, Narongchai Sangsa, Wirayut Silakun, Sunee Chayangsu, Noppol Buasi, Nicholas Day, Ganjana Lertmemongkolchai, Wasun Chantratita, T. Eoin West, Narisara Chantratita

**Affiliations:** a Faculty of Tropical Medicine, Department of Microbiology and Immunology, Mahidol University, Bangkok, Thailand; b Faculty of Infectious and Tropical Diseases, Department of Immunology and Infection, London School of Hygiene & Tropical Medicine, London, UK; c Faculty of Tropical Medicine, Mahidol-Oxford Tropical Medicine Research Unit, Mahidol University, Bangkok, Thailand; d Faculty of Public Health, Department of Microbiology, Mahidol University, Bangkok, Thailand; e Faculty of Medicine, Department of Surgery and Cancer, Imperial College London, London, UK; f Department of Tropical Medicine, Medical Microbiology, and Pharmacology, John A. Burns School of Medicine, University of Hawaii at Manoa, Honolulu, Hawaii, USA; g Department of Medicine, Udon Thani Hospital, Udon Thani, Thailand; h Department of Medicine, Nakhon Phanom Hospital, Nakhon Phanom, Thailand; i Department of Medicine, Mukdahan Hospital, Mukdahan, Thailand; j Department of Medicine, Roi Et Hospital, Roi Et, Thailand; k Department of Medicine, Buriram Hospital, Buriram, Thailand; l Department of Medicine, Surin Hospital, Surin, Thailand; m Department of Medicine, Sisaket Hospital, Sisaket, Thailand; n Centre for Tropical Medicine, Nuffield Department of Medicine, University of Oxford, Oxford, UK; o Faculty of Associated Medical Science, Department of Clinical Immunology, Khon Kaen University, Khon Kaen, Thailand; p The Centre for Research and Development of Medical Diagnostic Laboratories, Khon Kaen University, Khon Kaen, Thailand; q Faculty of Medicine Ramathibodi Hospital, Center for Medical Genomics, Mahidol University, Bangkok, Thailand; r Division of Pulmonary and Critical Care Medicine, Harborview Medical Center, University of Washington, Seattle, Washington, USA

**Keywords:** RNA-sequencing, transcriptomics, melioidosis, biomarkers, *Burkholderia pseudomallei*, outcome, immune response

## Abstract

Melioidosis is an often lethal tropical disease caused by the Gram-negative bacillus, *Burkholderia pseudomallei*. The study objective was to characterize transcriptomes in melioidosis patients and identify genes associated with outcome. Whole blood RNA-seq was performed in a discovery set of 29 melioidosis patients and 3 healthy controls. Transcriptomic profiles of patients who did not survive to 28 days were compared with patients who survived and healthy controls, showing 65 genes were significantly up-regulated and 218 were down-regulated in non-survivors compared to survivors. Up-regulated genes were involved in myeloid leukocyte activation, Toll-like receptor cascades and reactive oxygen species metabolic processes. Down-regulated genes were hematopoietic cell lineage, adaptive immune system and lymphocyte activation pathways. RT-qPCR was performed for 28 genes in a validation set of 60 melioidosis patients and 20 healthy controls, confirming differential expression. *IL1R2, GAS7, S100A9, IRAK3,* and *NFKBIA* were significantly higher in non-survivors compared with survivors (*P* < 0.005) and healthy controls (*P* < 0.0001). The AUROCC of these genes for mortality discrimination ranged from 0.80-0.88. In survivors, expression of *IL1R2*, *S100A9* and *IRAK3* genes decreased significantly over 28 days (*P* < 0.05). These findings augment our understanding of this severe infection, showing expression levels of specific genes are potential biomarkers to predict melioidosis outcomes.

## Introduction

Melioidosis is a severe infectious disease caused by *Burkholderia pseudomallei*, a Gram-negative bacterium and biothreat agent [1]. The disease is highly endemic in the tropics, particularly in Southeast Asia and northern Australia but reported cases are increasing globally. Melioidosis carries a mortality rate of 40% or higher in many endemic regions where resources are limited. This poor outcome from melioidosis has remained unchanged for many years [2,3]. Melioidosis is associated with several host factors, but diabetes is the major risk [4,5]. Pneumonia and bacteremia are the most common manifestations of disease; infections of these systems are frequently associated with septic shock and contribute to high mortality [2].

A comprehensive understanding of the individual response to infection is necessary to develop effective and targeted therapies. Additionally, biomarkers that predict outcome may be useful to guide patient management. Evaluation of the entire transcriptome of cells offers both the possibilities of characterizing pathways activated in disease and identifying potential biomarkers. In murine melioidosis, blood transcriptomic profiling reveals the regulation of many immune pathways, which reflect severity of disease [6] and can be used to identify a potential marker of acute lung infection [7]*.* Transcriptomic changes have been reported in human melioidosis during acute infection, highlighting the involvement of host immunity against infection [8]. Recent studies based on microarrays showed that blood transcriptional profiles can distinguish *B. pseudomallei* infection from sepsis caused by other microorganisms [9,10]. These studies suggest that these transcriptomic profiles may be useful in understanding the immune response during infection and serve as informative biomarkers of infection. RNA-sequencing (RNA-seq) is a unbiased approach and powerful tool to define the transcriptome [11]. However, to date, RNA-seq has not been used extensively to characterize human melioidosis. The aims of this study were to use RNA-seq (i) to analyze whole blood transcriptomic profiles of acute melioidosis patients to define biological pathways associated with death, and (ii) to identify host prognostic gene biomarkers that are associated with mortality.

## Methods

### Study design and patients

A prospective study of whole blood transcriptomic analyses in 97 individuals with melioidosis was conducted at seven hospitals in Northeast of Thailand: Udon Thani Hospital, Nakhon Phanom Hospital, Mukdahan Hospital, Roi Et Hospital, Buriram Hospital, Surin Hospial, and Sisaket Hospital. This study was part of a multi-centre study of patients aged ≥15 years who were culture-positive for *B. pseudomallei* from any type of clinical samples and admitted to the hospitals between January 2015 and December 2019. The inclusion and exclusion criteria were described previously [12]. *B. pseudomallei* were identified by biochemical tests and latex agglutination [13] at the microbiology laboratories of the hospitals and further confirmed by Matrix-Assisted Laser Desorption Ionization Mass Spectrometry (MALDI-TOF MS) as previously described [14]. Whole blood samples were collected at the time of enrolment (within 24 h of culture results, defined as day 0) and day 5, day 12, and day 28 after enrolment. Clinical information was obtained from the medical records. Mortality of patients was recorded at the hospitals or by phone calls for 28 days of follow-up.

Twenty-three healthy individuals aged ≥ 18 years were recruited from Udon Thani Hospital and Mukdahan hospital as baseline controls for discovery and validation data sets. Inclusion and exclusion criteria for these controls were previously described [15].

This study was designed by the process of 3 data sets as follows: discovery set, validation set, and follow-up set as described in Supplementary Figure 1.

### Ethical approval

The study was approved by the ethical committees of Faculty of Tropical Medicine, Mahidol University, Udon Thani Hospital, Nakhon Phanom Hospital, Mukdahan Hospital, Roi Et Hospital, Buriram Hospital, Surin Hospial, and Sisaket Hospital. Written informed consent was obtained from all participants or their representatives.

### Sample collection

Three milliliters of whole blood were collected from melioidosis patients and healthy controls into Tempus^TM^ Blood RNA Tubes (Thermo Fisher Scientific) and stored at −20°C or −80°C at the hospitals. The frozen samples were transported on dry ice to the laboratory in Bangkok for RNA extraction.

### RNA extraction

Total RNA was extracted from Tempus-stabilized blood using the MagMAX™ for Stabilized Blood Tubes RNA Isolation Kit (Life technologies). Total RNA concentration and its purity were assessed by determining the A260/280 and A260/230 ratios, respectively on the NanoDrop Spectrophotometer (Thermo Fisher Scientific). RNA integrity number (RIN) was assessed with the Agilent RNA 6000 Pico kit on 2100 Bioanalyzer (Agilent Technologies). Genomic DNA contamination was checked by RT-qPCR using primers for the Peptidylprolyl isomerase A (*PPIA)* gene [16].

### Library preparation for RNA-seq

Libraries were prepared from 50 ng of RNA per sample using Ion AmpliSeq™ Transcriptome Human Gene Expression Kit (Thermo Fisher Scientific). Targets of 20,802 genes were amplified with Ion AmpliSeq™ Transcriptome Human Gene Expression core panel (Life Technologies). The primer sequences were then digested, and DNA adaptors (Ion P1 Adaptor and Ion Xpress Barcode Adaptor, Life Technologies) were ligated to the targets. Adaptor ligated targets were purified using the Agencourt AMPure XP reagent (Beckman Coulter) and eluted into an amplification mix containing Platinum PCR SuperMix High Fidelity and Library Amplification Primer Mix (Life Technologies) for further amplification. Size-selection purification was performed using Agencourt AMPure XP reagent (Beckman Coulter). Amplicons were quantified using a Fragment Analyzer^TM^ instrument with a DNF-474 High Sensitivity NGS Fragment Analysis Kit (Advanced Analytical Technologies, INC.). Samples were then pooled together with four samples per pool and performed an emulsion PCR on the Ion Chef System using the Ion PI Hi-Q Chef Kit (Life Technologies). The emulsion PCR samples were loaded on Ion PI v3 chips and sequenced on an Ion Proton System using an Ion PI Hi-Q Sequencing 200 Kit chemistry (Life Technologies) to obtain approximately 200 bp read length.

### Transcriptomic data analysis

Sequencing data were generated using Torrent Suite Software version 5.4.0 with AmpliSeq RNA plugin (Thermo Fisher Scientific) and normalized using reads per million mapped reads (RPM) method. The normalized transcripts were analyzed using GeneSpring GX software version 14.9 (Agilent Technologies) to identify differentially expressed genes (DEGs) within the 10th -100^th^ percentile. One-way ANOVA was used to compare DEGs among non-survivors, survivors, and healthy controls. Moderated t-test was used to compare DEGs between non-survivors and survivors. An adjusted *P* value < 0.05 was deemed significant (Benjamini-Hochberg correction method). Functional analysis was derived using Metascape tool (http://metascape.org). Area under the receiver operating characteristic curves (AUROCC) were plotted using GraphPad Prism version 6.0.

DEGs were initially selected for validation based on fold change ≥ 2 and adjusted *P* value ≤ 0.05 between non-survivors and survivors.

### Quantitative reverse-transcriptase PCR (RT-qPCR)

Two-step RT-qPCR was used to quantitatively validate gene expression. Total RNA from whole blood was converted into cDNA using the iScript^TM^ cDNA Synthesis Kit (Bio-Rad). The amplification was performed in duplicate in a total volume of 10 μl containing 5 μl of iTaq Universal SYBR Green (Bio-Rad), 2 μl of 4 ng cDNA, 0.4 μl of 10 mM forward primer, 0.4 μl of 10 mM reverse primer, 2.2 μl of distilled water. The cycle conditions were as follows: 1 cycle of 95°C for 30s followed by 40 cycles of 95°C for 10s and 60°C for 30s. After amplification, melting curve analysis was carried out from 65°C to 95°C. Primers were designed using NCBI PrimerBlast (https://www.ncbi.nlm.nih.gov/tools/primer-blast/). All primer pairs are listed in Supplementary Table 1. Peptidylprolyl isomerase A (*PPIA*), Human large ribosomal protein P0 (*RPLP0*), and Tata-box binding protein (*TBP*) were used as reference genes for calculating the relative expression levels of other genes [16] The expression levels were calculated by using the 2^−ΔCt^ method, where ΔCt = mean Ct of target gene – mean Ct of the three reference genes.

### Statistical analysis

Mann–Whitney or Kruskal–Wallis tests followed by Dunn’s multiple comparison tests correction were used to test the difference in gene expressions among subject groups. Mean, median, interquartile range (IQR), standard deviation (SD), area under the receiver operating characteristic curve (AUROCC) values and 95% confidence intervals (CI) were assessed using Prism 6 (GraphPad Software). The classification accuracy of the 12 gene signature was determined using the randomForest machine learning R package (v. 4.16) [17] applied to the qRT-PCR data. The AUROCC curve was visualised using the pROC package (v. 1.10).

## Results

### Whole blood transcriptomic profiles of survivors and non-survivors

**Figure 1.**
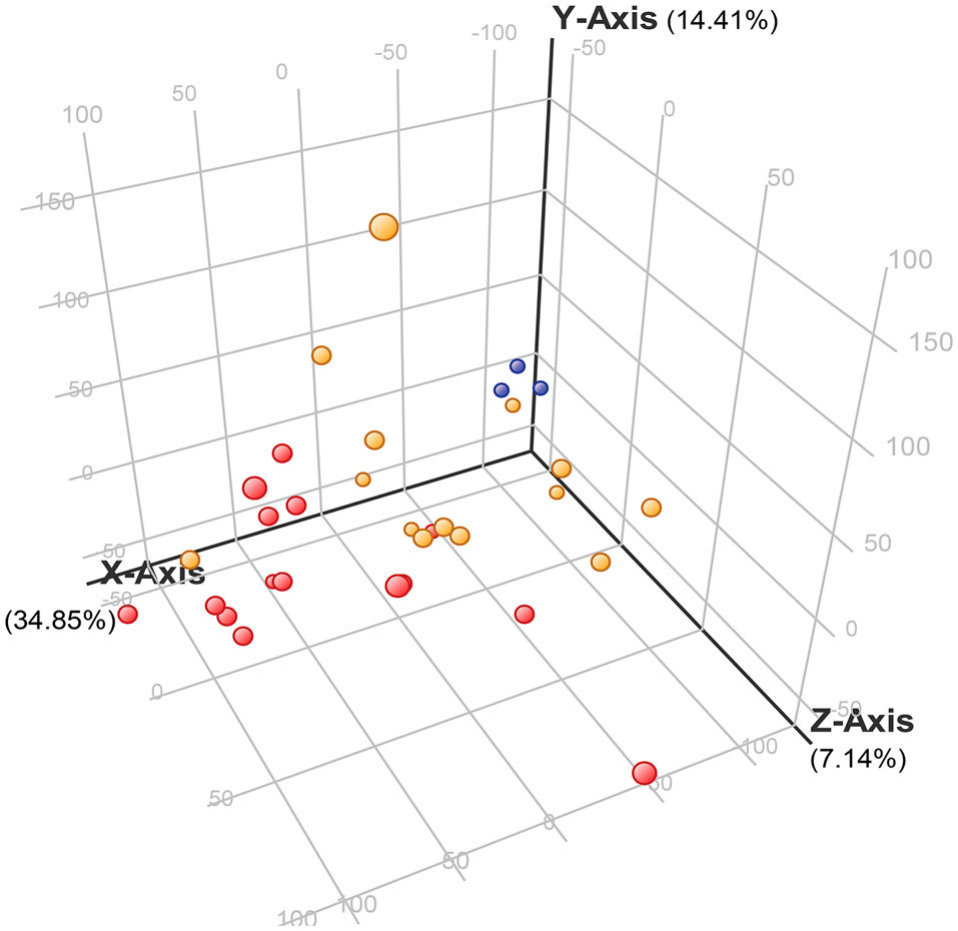
Three-dimensional principal component analysis (3D-PCA) of differentially expressed genes among non-survivors and survivors and healthy controls. One point per subject in yellow, red, and light blue, represents groups of melioidosis patients who survived (*n* = 14) and did not survive (*n* = 15), and healthy controls (*n* = 3), respectively. Each axis shows percent variation explained by each group.

To identify genes associated with mortality, we performed whole blood transcriptomic analysis of a discovery set consisting of 29 Thai melioidosis patients, fourteen of whom survived and fifteen of whom died within 28 days, and 3 healthy controls. The clinical characteristics of the patients are shown in Table 1. The quality of 32 RNA samples were analyzed for integrity and read count/mapped read numbers. Overall average RNA integrity numbers (RIN) of 6.0-8.6, average OD ratios 260/280 > 1.8, 260/230 < 1, and average of 22 million reads with mapping rate of >58% were achieved from each cDNA library. Out of 20,802 genes, 18,713 genes with expression values between 10th - 100th percentiles were further analyzed using one-way ANOVA and 5,189 genes were statistically different among groups as shown in three dimensional principal component analysis (3D-PCA) plots (Figure 1).

**Table 1.** Characteristics of melioidosis patients and healthy controls.

	Discovery cohort		Validation cohort		Follow-up cohort (*n*=8)	Healthy control (*n* = 23)
Characteristics	Non-survivors (*n* = 15)	Survivors (*n* = 14)	Non-survivors (*n* = 30)	Survivors (*n* = 30)		
**Mean age in years (range)**	57 (36–81)	51 (28–74)	62	60	50 (32–70)	43
			(45–84)	(34–80)		(28–68)
**Male (%)**	11 (73%)	10 (71%)	26 (87%)	21 (70%)	8 (100%)	14 (61%)
**Comorbidity**						
Diabetes (%)	9 (60%)	7 (50%)	18 (60%)	17 (57%)	7 (88%)	–
Alcoholism (%)	3 (20%)	6 (43%)	7 (23%)	10 (33%)	3 (38%)	–
Kidney disease (%)	2 (13%)	1 (7%)	5 (17%)	3 (10%)	5 (63%)	–
Hypertension (%)	5 (33%)	2 (14%)	13 (43%)	7 (23%)	3 (38%)	–
Thalassemia (%)	–	2 (14%)	–	1 (3%)	–	–
Cancer (%)	2 (13%)	–	2 (7%)	1 (3%)	–	–
None (%)	3 (20%)	1 (7%)	4 (13%)	4 (13%)	–	23 (100%)
**Clinical symptom**						
Bacteremia (%)	14 (93%)	8 (57%)	28 (93%)	23 (77%)	7 (88%)	–
**Fever**						
<15 days (%)	14 (93%)	11 (79%)	28 (93%)	23 (77%)	7 (88%)	–
≥15 days (%)	1 (7%)	3 (21%)	2 (7%)	7 (23%)	1 (13%)	–

**Figure 2.**
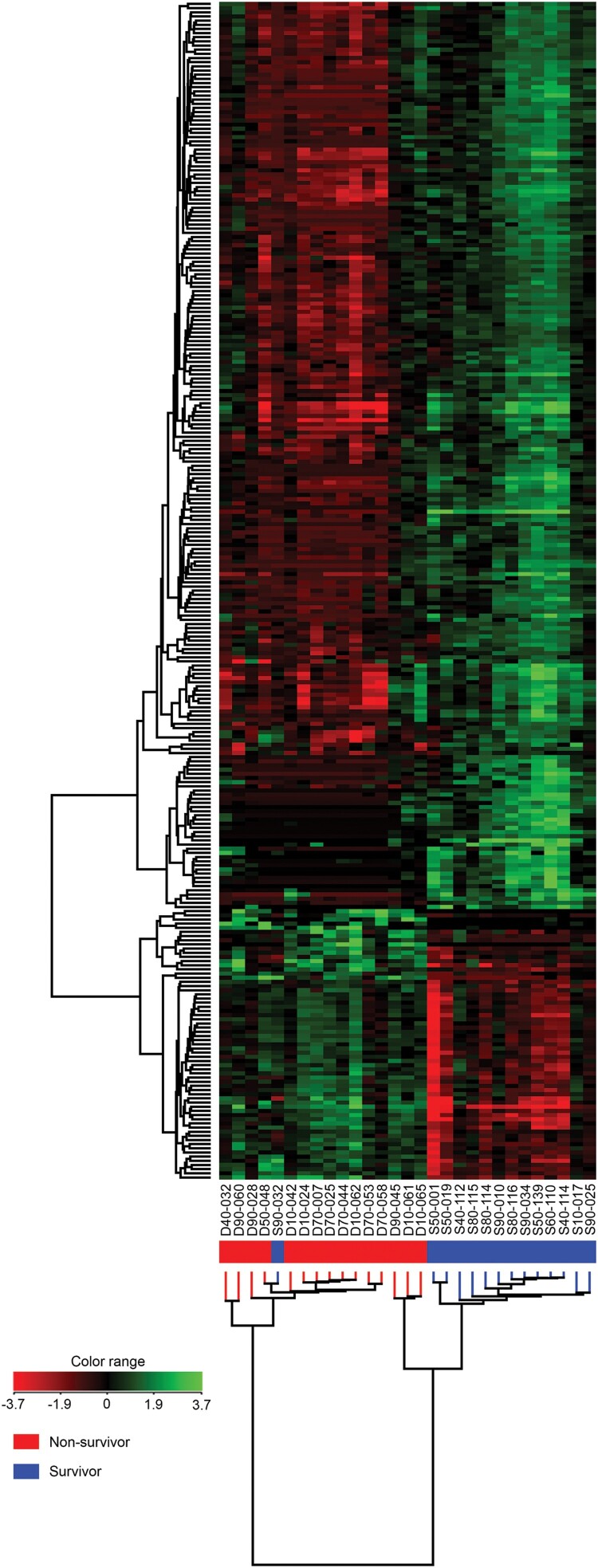
Hierarchical clustering analysis of 283 differentially expressed genes (DEGs) in whole blood of surviving and non-surviving melioidosis patients. High expression of genes is shown in green whereas low expression of genes is shown in red. Each column represents individual subjects and each row in the figure represents one altered gene that significantly expressed at *P* ≤ 0.05 and fold change ≥ 2. Subjects from our study are melioidosis survivors (*n* = 14), melioidosis non-survivors (*n* = 15).

**Figure 3.**
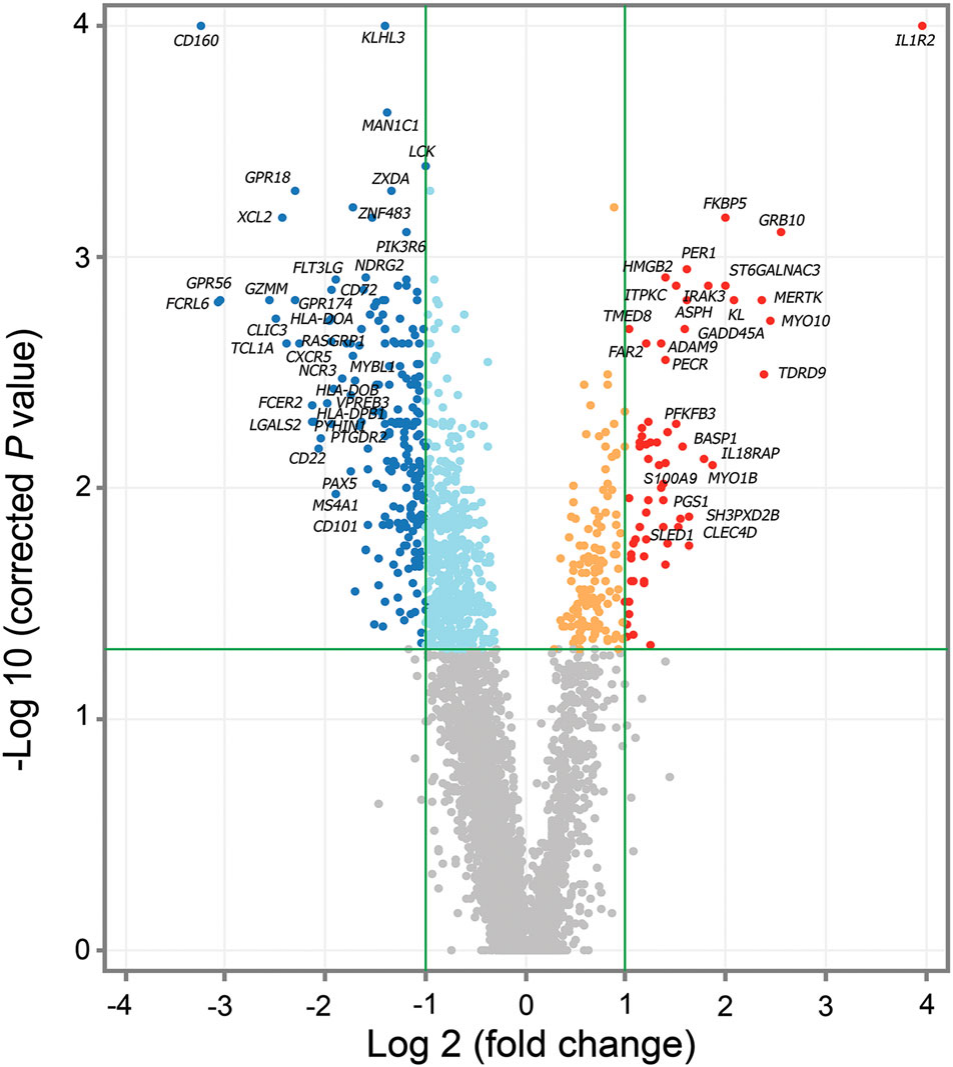
Differential expression analysis of survivors compared to non-survivors at the time of diagnosis (day 0). Gene expression profile of patients with melioidosis that survived after 28 days (*n* = 14) compared to patients that did not survive (*n* = 15). Color indicates statistically significant genes (adjusted *P* value ≤ 0.05, correction method = Benjamini-Hochberg), dark blue: down-regulated genes ≥ 2 fold change, dark red: up-regulated genes ≥ 2 fold change with grey corresponding to genes showing no expression change.

Analysis of differentially expressed genes (DEGs) between non-survivors and survivors performed using the moderated t-test method identified 283 DEGs. Hierarchical cluster analysis of these genes was generated by GeneSpring (Figure 2). Whole blood of non-survivors presented more down-regulated genes compared to survivors (fold change ≥ 2). RNA-seq data of 65 up-regulated genes and 218 down-regulated genes with *P* value ≤ 0.05 and fold change ≥ 2 are shown in Supplementary Table 2. In comparison to melioidosis patients who survived, the fold changes of up-regulated genes in non-survivors ranged between 2.00–15.72 and *P* value = 1.70 × 10^−3^ to 5.47 × 10^−9^. The fold change of down-regulated genes ranged between 2.00–9.42 and *P* value = 9.50 × 10^−5^ to 2.54 × 10^−9^. The volcano plot in Figure 3 shows the distribution and relationship between fold change and *P* value of 65 up-regulated genes and 218 down-regulated genes in non-survivors in relation to survivors.

## Functional enrichment analysis of DEGs between survivors and non-survivors

**Figure 4.**
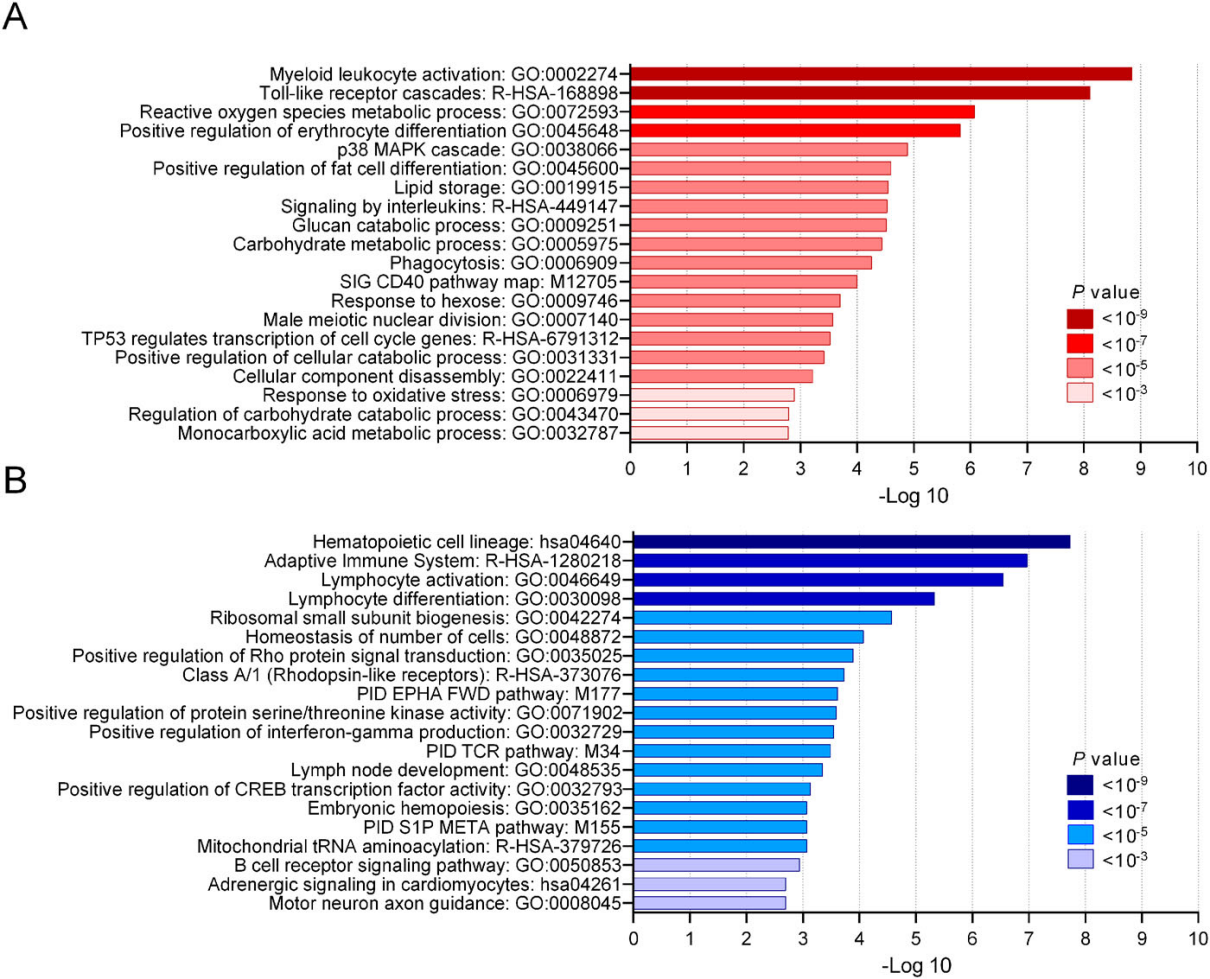
Functional enrichment analysis of DEGs in non-surviving melioidosis patients compared with patients that survived. (A) Top 20 enriched terms of 65 up-regulated genes in non-surviving melioidosis patients. (B) Top 20 enriched terms of 218 down-regulated genes in non-surviving melioidosis. Saturation of color corresponds to *P* values.

In order to gain insight into the biological function of DEGs, the genes found significantly differential expressed (65 up-regulated and 218 down-regulated) between survivors and non-survivors were analyzed using the Metascape tool. The analysis was based on combined datasets for enrichment analysis, including gene ontology, KEGG pathways, reactome gene sets, canonical pathways, and CORUM complexes. The data in Figure 4 shows that the significant DEGs were involved in functions of host immune response (n = 7), stress response (n = 6), cell development (n = 35), signaling transduction (n = 23), catabolic process (n = 16), and metabolic process (n = 24). The significant 65 up-regulated DEGs in non-survivors were involved in myeloid leukocyte activation (n = 14), Toll-like receptor cascades (n = 8), and reactive oxygen species metabolic processes (n = 8) (Figure 4A) while the majority of 218 down-regulated genes set in non-survivors were hematopoietic cell lineage (n = 10), adaptive immune system (n = 24) and lymphocyte activation (n = 23) (Figure 4B). Gene names and details of each functional group are shown in Supplementary Table 3.

## Pathway analysis of DEGs between melioidosis survivors and non-survivors

To gain better understanding of the underlying mechanisms of the 283 altered genes in non-survivors compared to survivors, we performed KEGG pathway analysis. Interestingly, KEGG identified six pathways in immunological response that were associated with 65 up-regulated genes (Supplementary Table 4). These included pathways of Toll-like receptor signalling, Th17 cell differentiation, MAPK, IL-17 signalling, FoxO signalling, HIF-1 signalling. Moreover, KEGG identified seven pathways in immunological response that were associated with 218 down-regulated genes. These included hematopoietic cell lineage, cell adhesion molecules (CAMs), intestinal immune network for IgA production, Th1 and Th2 cell differentiation, Th17 cell differentiation, antigen processing and presentation and B cell receptor signalling pathway.

## RT-qPCR validation of DEGs to predict mortality in melioidosis

**Figure 5.**
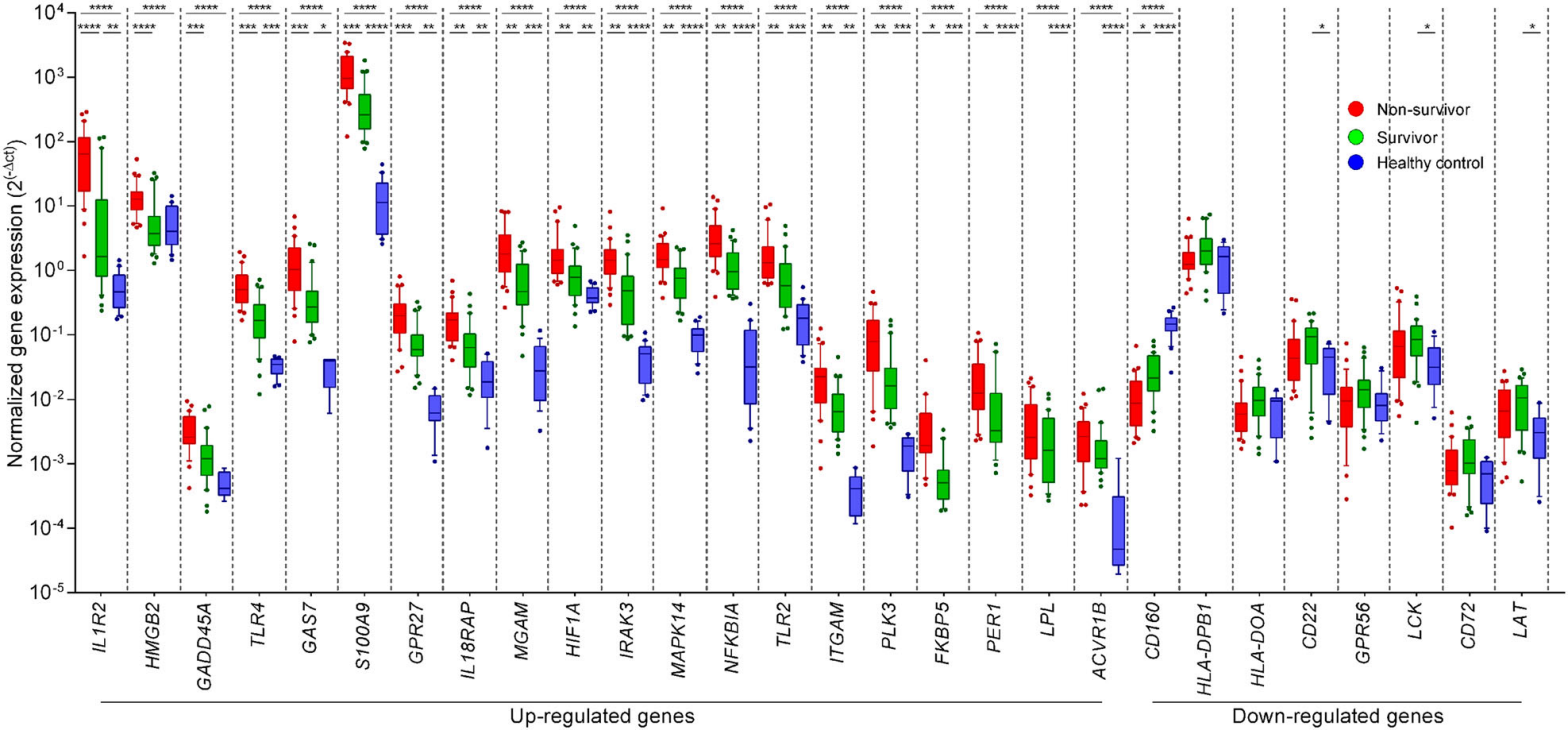
Validation of the differential expression analysis of 28 DEGs in whole blood from melioidosis patients. Genes that were found to be differentially expressed in patients with melioidosis that did not survive and survived were validated with real-time qPCR. The Kruskal–Wallis test was performed for comparing three groups. Subjects from our study were melioidosis survivors (*n* = 30), melioidosis non-survivors (*n* = 30), and healthy controls (*n* = 20). *P ≤ 0.05, **P ≤ 0.01, ***P ≤ 0.005, ****P ≤ 0.0001.

Twenty-eight DEGs were manually selected to confirm the expression by RT-qPCR in a validation set of 30 non-survivors, 30 survivors and 20 healthy controls. The DEGs were selected according to (i) their degree of alteration (fold changes and *P* value) (Supplementary Table 2) and (ii) their functions related with immunological responses (Supplementary Table 4). These DEGs included 20 up-regulated genes and 8 down-regulated. RT-qPCR results in the validation set confirmed significantly higher expression in non-survivors compared with survivors and healthy controls for 16 of the 20 up-regulated genes and 1 of the 8 down-regulated genes, respectively (Figure 5 and Supplementary Table 5). RT-qPCR in the validation set confirmed significantly lower expression in non-survivors compared with survivors (*P* = 0.016) and healthy controls (*P* < 0.0001) for 1 of 8 down-regulated genes: *CD160*.

### ROC assessment of gene expression as predictive markers for mortality

**Figure 6.**
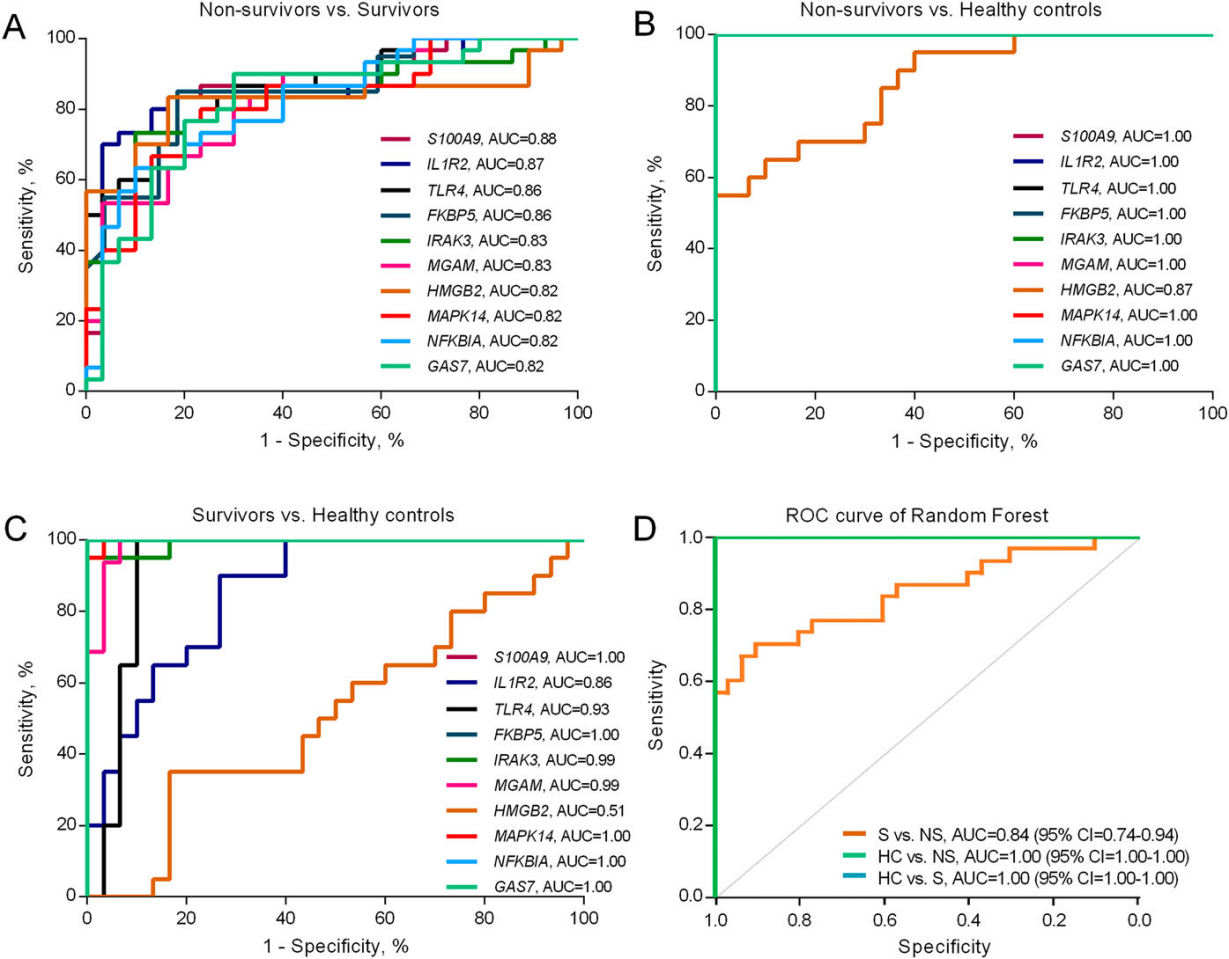
Area under the receiver operating characteristic curve (AUROCC) of DEGs in discrimination among non-survivors, survivors and healthy controls. (A) AUROCC of 10 DEGs between non-survivors versus survivors. (B) AUROCC of 10 DEGs between non-survivors versus healthy controls. (C) AUROCC of 10 DEGs between survivors versus healthy controls. (D). Random Forest model of a combined gene signature discriminates survivors and non-survivors. The 12 genes which individually discriminated clinical groups with AUROCC > 0.80 in qRT-PCR were combined to create a single model, which was used to classify the separation between survivors (S), non-survivors (NS) and healthy controls (HC) in the qRT-PCR dataset.

Receiver operating characteristic (ROC) curves were constructed based on the RT-qPCR results from the validation set of melioidosis patients to examine the classification accuracy of each DEG for distinguishing between non-survivors and survivors (Figure 6A-C). The highest area under the ROC (AUROCC) were obtained from the genes listed in Supplementary Table 6. Among these, *S100A9* showed the highest AUROCC value (0.88) followed by *IL1R2* (0.87) and *TLR4* (0.86). The down-regulated gene with the highest AUROCC was *CD160* (0.77). A combined signature of the expression of the 12 genes with best individual discriminatory ability was able to classify the non-survivors from the survivors in a Random Forest model (AUROCC 0.85, CI = 0.74–0.94), and completely discriminated the melioidosis patients from the healthy controls (Figure 6D).

## Trajectory of gene expression profiles in survivors after enrolment

**Figure 7.**
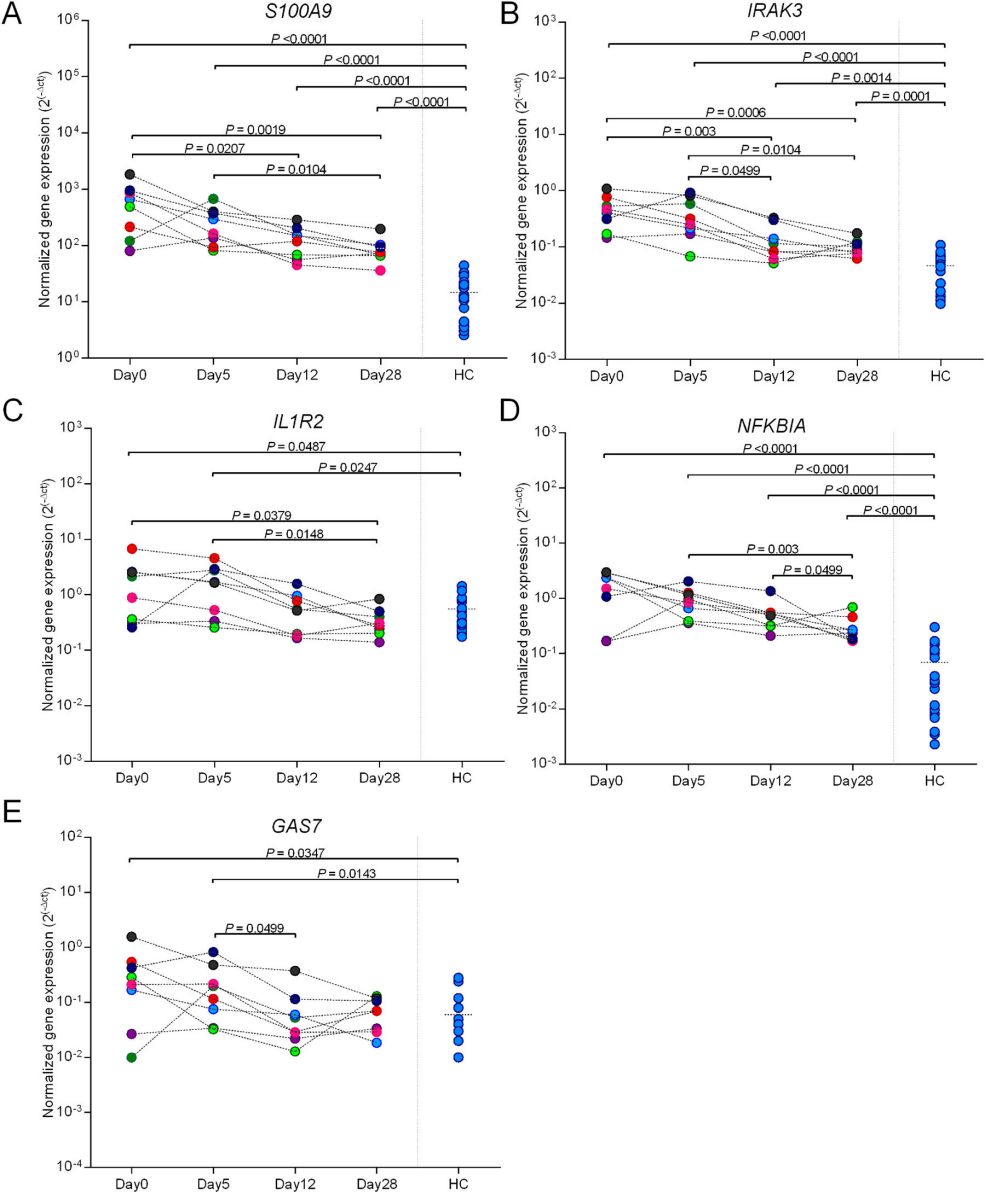
One month follow-up of *S100A9, IRAK3, IL1R2, GAP7,* and *NFKBIA* in surviving melioidosis patients over the course of illness. Whole blood samples from melioidosis survivors (*n* = 8) were collected at the various times from diagnosis (day 0, day 5, day 12, and day 28). The *P-*values were calculated by Mann–Whitney test. Data of healthy individuals were plotted as the controls.

Five up-regulated DEGs (*S100A9, IL1R2, IRAK3, NFKBIA* and *GAS7*) were selected based on AUROCC ≥ 0.82 and whether the genes have secretory functions of proteins as they may be better suited to a point-of-care assay. Gene expression was measured by RT-qPCR in survivors (n = 8) at day 0, day 5, day 12, and day 28 to test whether expression decreases as patients recovered. The trend of gene expression at day 0, day 5, day 12, and day 28 were determined by calculating the fold change reduction. None of the five genes had major changes in expression at day 5 but *S100A9, IRAK3* and *IL1R2* subsequently had decreased expression over time as patients recovered (Figure 7 and Supplementary Table 7). Expression of *S100A9, IRAK3, IL1R2* and *NFKBIA* significantly decreased at day 28 relative to day 5. Expression of *S100A9*, *IRAK3*, and *NFKBIA* in patients decreased at day 28 but did not reach to the expression level of healthy controls (*P* < 0.0001). However, expression of *IL1R2* and *GAS7* rapidly decreased to the same level of healthy controls and did not change further after day 12 (*P* < 0.05). The mean fold changes (day 28/day 5) for gene expression of 8 individual patients and 95% CI are shown in Supplementary Table 8.

## Discussion

Our study demonstrated that the whole blood transcriptome of melioidosis patients who survived was distinguishable from non-survivors, with 283 DEGs significantly associated with mortality. The majority of these DEGs were related to the immune response, cellular functions and metabolism. Twenty-eight DEGs were selected by functional enrichment and pathway analyses and RT-qPCR of these genes in a validation cohort confirmed 16 up-regulated and 1 down-regulated gene associated with mortality. ROC analyses of the validation set identified the 15 most predictive genes. Subsequent RT-qPCR of four selected genes (*S100A9, IRAK3, IL1R2,* and *NFKBIA*) in surviving patients followed over time demonstrated a trajectory expression profile with decreased differential expression by day 12 and day 28 after enrolment.

Genes of melioidosis patients associated with death include *IL1R2, IRAK3, IL18RAP, MGAM, LPL, HGMB2, S100A9, GAS7, NFKBIA, TLR2, TLR4, MAPK14, GPR27, HIF1A,* and *ITGAM.* Many of these genes or their proteins have been reported in related studies. Elevation of *IL1R2* expression and soluble *IL1R2* concentrations are correlated with severity of *Escherichia coli* and *Staphylococcus aureus* infections [18]. Increased expression levels of the *IRAK3* gene are correlated with the development of acute lung injury in patients with severe sepsis [19]. In melioidosis, Wiersinga et al. reported up-regulation of *IRAK3* is related to attenuated capacity of monocytes to respond to *B. pseudomallei* stimulation and this coincided with mortality [20]. In parallel to our study, a recent study reported that extracellular S100A8 and S100A9 (S100A8/A9), a Ca^2+^ sensor in cytoskeleton rearrangement and arachidonic acid metabolism, are the key mediators of sepsis secreted from neutrophils and monocytes during inflammation [21]. The S100A9 serve as damage associated molecular patterns and induce pro-inflammatory cytokine expression and secretion via toll-like receptor 4 (TLR4) activation [22,23]. Increasing evidence supports that *NFKBIA*-mediated inflammation is linked to susceptibility to infectious and inflammatory diseases [24–26]. A report demonstrated an up-regulation of *NFKBIA* expression in mouse macrophages in response to *B. pseudomallei* infection [27] and our data confirmed that increased *NFKBIA* expression is associated with fatality in melioidosis patients.

A recent study suggests that *HLA-DPA1* and *-DRB3* are under-expressed in whole blood of sepsis patients caused by *B. pseudomallei*, which distinguished melioidosis from sepsis caused by other organisms [9]. In addition, we found *HLA-DPB1* was down-regulated in non-survivors in our discovery cohort. Our data also revealed that non-survivors had reduced expression of *HLA-DPB1, HLA-DOA, HLA-DOB*, and *HLA-DRA* representing MHC class II molecules, which are important for antigen presentation. Our results in melioidosis are similar to the results of other studies [28–30] suggesting that non-surviving patients with severe sepsis from melioidosis or other infections exhibit decreased MHC class II expression and that can contribute to persistent failure of T cell activation [31,32]. We did not observe the changes of these MHC class I at transcriptional levels. However, Dunachie et al. showed the presence of MHC class I genes, *HLA-B46*, and HLA-C*01 was associated with an increased mortality in an acute melioidosis cohort [8].

Enrichment analysis demonstrated a number of GO terms, including the up-regulation of myeloid leukocyte activation and down-regulation of lymphocyte activation in non-survivors compared with survivors. KEGG pathway analysis revealed many up-regulated genes involved in signal transduction pathways associated with severe melioidosis. Among these, TLRs are known to recognize *B. pseudomallei* LPS and initiate inflammation [33–36] and acute septic melioidosis patients had increased expression of many TLRs in leukocytes [34]. The activation of MAPK signaling and Th17 pathway in melioidosis patients have also been demonstrated in previous studies [37–40]. Multiple signaling pathways were down-regulated in severe melioidosis suggesting that prolonged bacterial persistence exacerbates inflammatory responses that may lead to immune exhaustion, immune suppression, and poor outcome of the disease.

Expression of several genes, assayed on day 0, had high mortality discrimination, including *S100A9* and *IL1R2.* Notably, expression of these genes decreased significantly in surviving patients by day 12, suggesting that the gene expression tracks with clinical condition. Therefore, these genes and their encoded proteins could be considered as candidate biomarkers for predicting clinical outcomes in patients with melioidosis, and deserve further study in comparison to other clinical and biological prediction tools.

Strengths of our study were the multi-center design, prospective subject enrolment and sample collection, serial sampling over time in a subset of patients, and validation of selected findings. Some limitations are the relatively small number of samples in the discovery cohort, enrolment into our study only after the diagnosis of melioidosis was confirmed (rather than at the time of admission to hospital), and validation of only a subset of genes.

In conclusion, our findings provide new knowledge about transcriptional host responses in circulating leukocytes from hospitalized melioidosis patients and suggest several candidate biomarkers for further study. These data are important to ongoing efforts to reduce the burden of this often severe infection.

## Supplementary Material

Transcriptome_Supplementary_Tables_EMI_10Sep20_2.docx

Supplementary_Figure_1.tif
