# Supplementary material for: Blood transcriptomics to characterize key biological pathways and identify biomarkers for predicting mortality in melioidosis
Source: Emerg Microbes Infect. 2021 Jan 17;10(1):8–18. doi: 10.1080/22221751.2020.1858176 (PMC7832033; doi:10.1080/22221751.2020.1858176)
Supplement: Transcriptome_Supplementary_Tables_EMI_10Sep20_2.docx [file TEMI_A_1858176_SM9797.docx]

**Supplementary Table 1.** Oligonucleotide primers used for quantitative RT-PCR (RT-qPCR).

| **Gene symbol** | **Gene description** | **Primer sequences (5’-3’)** | **Amplicon size (bp)** | **TM (°C)** | **References** |
| --- | --- | --- | --- | --- | --- |
| *PPIA* | Peptidylprolyl isomerase A | S_GCTGGACCCAACACAAATGG | 86 | 59.68 | [1] |
|  |  | A_TTGCCAAACACCACATGCTT |  | 59.17 |  |
| *TBP* | Tata-box binding protein | S_ATGGTGGGGAGCTGTGATGT | 101 | 61.21 | [1] |
|  |  | A_AAACCAGGAAATAACTCTGGCTCA |  | 60.20 |  |
| *RPLP0* | Human large ribosomal protein P0 | S_GCTTCCTGGAGGGTGTCC | 105 | 59.33 | [1] |
|  |  | A_GGACTCGTTTGTACCCGTTG |  | 58.86 |  |
| *TLR4* | Toll like receptor 4 | F_CAACCATTTGCCAGACACCA | 143 | 58.96 | This study |
|  |  | R_ACGGGAAGCACAACCATCTA |  | 59.02 |  |
| *TLR2* | Toll like receptor 2 | F_TGCAAGCAGGATCCAAAGGA | 111 | 59.59 | This study |
|  |  | R_CAAGACCCACACCATCCACA |  | 59.89 |  |
| *CD160* | CD160 molecule | F_GCTTTGTAAGCCTTGTGCCA | 119 | 59.33 | This study |
|  |  | R_CCTGTGCCCTGTTGCATTCT |  | 60.90 |  |
| *IL1R2* | Interleukin 1 receptor type 2 | F_TGTGCTGGCCCCACTTTC | 101 | 60.20 | [2] |
|  |  | R_GCACAGTCAGACCATCTGCTTT |  | 61.13 |  |
| *S100A9* | S100 calcium binding protein A9 | F_TGGAGGACCTGGACACAAATG | 109 | 59.93 | [3] |
|  |  | R_TCGTCACCCTCGTGCATCTT |  | 61.53 |  |
| *NFKBIA* | NFKB inhibitor alpha | F_CTCCGAGACTTTCGAGGAAATAC | 135 | 58.65 | [4] |
|  |  | R_GCCATTGAAGTTGGTAGCCTTCA |  | 61.37 |  |
| *HIF1A* | Hypoxia inducible factor 1 subunit alpha | F_CATAAAGTCTGCAACATGGAAGGT | 148 | 59.54 | [5] |
|  |  | R_ATTTGATGGGTGAGGAATGGGTT |  | 60.25 |  |
| *PLK3* | Polo like kinase 3 | F_TCACTGGGCTGTGTCATGTA | 96 | 58.65 | [6] |
|  |  | R_GTGAACCTGCTTGATGCAG |  | 56.92 |  |
| *GADD45A* | Growth arrest and DNA damage inducible alpha | F_AGAAGACCGAAAGCGACCC | 131 | 59.71 | This study |
|  |  | R_GTTGATGTCGTTCTCGCAGC |  | 59.91 |  |
| *CD22* | CD22 molecule | F_GCCAGAGCTTCTTTGTGAGG | 182 | 58.84 | [7] |
|  |  | R_GGGAGGTCTCTGCATCTCTG |  | 59.25 |  |
| *HLA-DOA* | Major histocompatibility complex, class II, DO alpha | F_TTTGCCCGCTTTGACCCGCA | 118 | 65.99 | This study |
|  |  | R_TCACCCGTGGAGGCACGTTG |  | 65.10 |  |
| *LCK* | LCK proto-oncogene, Src family tyrosine kinase | F_TGCCATTATCCCATAGTCCCA | 95 | 58.29 | This study |
|  |  | R_GAGCCTTCGTAGGTAACCAGT |  | 59.18 |  |
| *LAT* | Linker for activation of T cells | F_CTACCCACCTGTCACCTCCT | 129 | 60.25 | This study |
|  |  | R_CTGTTGGCACCATCAGAATC |  | 56.78 |  |
| *HLA-DPB1* | Major histocompatibility complex, class II, DP beta 1 | F_CCTGGTGATGCTGGAAATG | 105 | 56.26 | This study |
|  |  | R_GACTGTGCCTTCCACTCCA |  | 59.25 |  |
| *CD72* | CD 72 molecule | F_CAGCTCCGCCTCAAGATAAC | 177 | 58.42 | This study |
|  |  | R_TTGCAAGGTCTCCTTCGTCT |  | 58.95 |  |
| *IRAK3* | Interleukin 1 receptor associated kinase 3 | F_CAGCCAGTCTGAGGTTATGTTT | 110 | 58.32 | [8] |
|  |  | R_TTGGGAACCAACTTTCTTCACA |  | 58.30 |  |
| *ITGAM* | Integrin subunit alpha M | F_ATGCAGAAACAGGGATGGGA | 71 | 59.00 | This study |
|  |  | R_GATAGCAGCGTGGAACCAAG |  | 58.99 |  |
| *KL* | Klotho | F_ACTGGATCACCATCGACAACCC | 192 | 62.32 | This study |
|  |  | R_CAATGGACACCTGACCTCCCT |  | 61.46 |  |
| *FKBP5* | FKBP prolyl isomerase 5 | F_GAGTTACATCCCCCATGCCAA | 149 | 60.06 | This study |
|  |  | R_GGGGATTGTCGCTTCGTAGT |  | 59.82 |  |
| *IL18RAP* | Interleukin 18 receptor accessory protein | F_CGTTCAGATACAAAAGCTGGCAGT | 125 | 61.86 | This study |
|  |  | R_TCCCTTTCAGTTGGTCAAGGCT |  | 61.83 |  |
| *PER1* | Period circadian regulator 1 | F_GAGGACACTCCTGCGACCAG | 192 | 62.22 | This study |
|  |  | R_TCCCCCATCAGCCCCTTCTA |  | 61.91 |  |
| *MGAM* | Maltase-glucoamylase | F_CACCCTCCCTACATGCCACA | 95 | 61.56 | This study |
|  |  | R_GAGCCGTCTGGGAGGATCTG |  | 61.74 |  |
| *HMGB2* | High mobility group box 2 | F_CCCTGGCCTATCCATTGGGG | 176 | 62.09 | This study |
|  |  | R_CAGGGCCCTTCTTTCCTGCT |  | 62.15 |  |
| *GAS7* | Growth arrest specific 7 | F_TGCGACTACTTCTGGGCTGA | 102 | 60.90 | This study |
|  |  | R_CTGCATTTGTTTGCCCTTCA |  | 57.47 |  |
| *MAPK14* | Mitogen-activated protein kinase 14 | F_GGGGCTGAGCTTTTGAAGAAA | 180 | 59.04 | This study |
|  |  | R_GGCTTGGGCCGCTGTAATTC |  | 62.00 |  |
| *GPR27* | G protein-coupled receptor 27 | F_GCAAGATGTTCTACGCCGTCA | 194 | 61.00 | This study |
|  |  | R_GTCCCTCAGCTCCCTGTTGAA |  | 61.72 |  |
| *LPL* | Lipoprotein lipase | F_ACGGGCTCAGGAGCATTACC | 142 | 61.97 | This study |
|  |  | R_GGCTCCAAGGCTGTATCCCA |  | 61.64 |  |
| *ACVR1B* | Activin A receptor type 1B | F_CAGCAGAACCTTGGCGGTTTA | 85 | 61.15 | [9] |
|  |  | R_GTTGGCAGATCCCAGAGGCTAC |  | 62.70 |  |

**Supplementary Table 2.** Differentially expressed genes in whole blood of melioidosis patients who were survived and died. The data show 65 up-regulated genes and 218 down-regulated genes in non-survivors.

| **Gene** | **Description** | **Fold change** | ***P* value** | **Regulation** |
| --- | --- | --- | --- | --- |
| *IL1R2* | Interleukin 1 receptor type 2 | 15.72 | 5.5E-09 | up |
| *GRB10* | Growth factor receptor bound protein 10 | 5.88 | 9.0E-07 | up |
| *MYO10* | Myosin X | 5.48 | 7.6E-06 | up |
| *TDRD9* | Tudor domain containing 9 | 5.25 | 2.2E-05 | up |
| *MERTK* | MER proto-oncogene, tyrosine kinase | 5.16 | 3.7E-06 | up |
| *KL* | Klotho | 4.27 | 3.8E-06 | up |
| *ST6GALNAC3* | ST6 N-acetylgalactosaminide alpha-2,6-sialyltransferase 3 | 4.02 | 2.4E-06 | up |
| *FKBP5* | FKBP prolyl isomerase 5 | 4.02 | 6.3E-07 | up |
| *MYO1B* | Myosin IB | 3.67 | 1.3E-04 | up |
| *IRAK3* | Interleukin 1 receptor associated kinase 3 | 3.53 | 2.6E-06 | up |
| *IL18RAP* | Interleukin 18 receptor accessory protein | 3.43 | 1.2E-04 | up |
| *SH3PXD2B* | SH3 and PX domains 2B | 3.10 | 3.5E-04 | up |
| *CLEC4D* | C-type lectin domain family 4 member D | 3.10 | 6.3E-04 | up |
| *PER1* | Period circadian regulator 1 | 3.06 | 1.4E-06 | up |
| *ASPH* | Aspartate beta-hydroxylase | 3.04 | 4.6E-06 | up |
| *GADD45A* | Growth arrest and DNA damage inducible alpha | 3.01 | 1.0E-05 | up |
| *BASP1* | Brain abundant membrane attached signal protein 1 | 2.96 | 9.8E-05 | up |
| *PGS1* | Phosphatidylglycerophosphate synthase 1 | 2.93 | 3.7E-04 | up |
| *SLED1* | Proteoglycan 3, pro eosinophil major basic protein 2 pseudogene | 2.87 | 4.6E-04 | up |
| *ITPKC* | Inositol-trisphosphate 3-kinase C | 2.86 | 2.6E-06 | up |
| *PFKFB3* | 6-phosphofructo-2-kinase/fructose-2,6-biphosphatase 3 | 2.86 | 6.3E-05 | up |
| *SLC26A6* | Solute carrier family 26 member 6 | 2.68 | 7.4E-05 | up |
| *SCN5A* | Sodium voltage-gated channel alpha subunit 5 | 2.68 | 6.0E-04 | up |
| *PECR* | Peroxisomal trans-2-enoyl-CoA reductase | 2.66 | 1.8E-05 | up |
| *MGAM* | Maltase-glucoamylase | 2.65 | 9.5E-04 | up |
| *SLC2A3* | Solute carrier family 2 member 3 | 2.64 | 1.3E-04 | up |
| *HMGB2* | High mobility group box 2 | 2.64 | 1.6E-06 | up |
| *SYCP2* | Synaptonemal complex protein 2 | 2.62 | 4.6E-04 | up |
| *SULT1B1* | Sulfotransferase family 1B member 1 | 2.59 | 2.6E-04 | up |
| *S100A9* | S100 calcium binding protein A9 | 2.59 | 1.9E-04 | up |
| *ADAM9* | ADAM metallopeptidase domain 9 | 2.57 | 1.3E-05 | up |
| *GAS7* | Growth arrest specific 7 | 2.55 | 2.1E-04 | up |
| *NFKBIA* | NFKB inhibitor alpha | 2.52 | 1.4E-04 | up |
| *ARMC12* | Armadillo repeat containing 12 | 2.48 | 9.1E-05 | up |
| *TLR2* | Toll like receptor 2 | 2.37 | 8.9E-05 | up |
| *CCNA1* | Cyclin A1 | 2.37 | 3.4E-03 | up |
| *RALGAPA2* | Ral GTPase activating protein catalytic alpha subunit 2 | 2.35 | 2.7E-04 | up |
| *RNF144B* | Ring finger protein 144B | 2.35 | 1.2E-04 | up |
| *KRT8* | Keratin 8 | 2.33 | 5.3E-05 | up |
| *TLR4* | Toll like receptor 4 | 2.32 | 3.2E-04 | up |
| *FAR2* | Fatty acyl-CoA reductase 2 | 2.31 | 1.3E-05 | up |
| *GNG10* | G protein subunit gamma 10 | 2.31 | 5.6E-04 | up |
| *KLF7* | Kruppel like factor 7 | 2.30 | 9.3E-05 | up |
| *PLK3* | Polo like kinase 3 | 2.29 | 7.8E-04 | up |
| *LHX4* | LIM homeobox 4 | 2.29 | 1.2E-03 | up |
| *ZNF438* | Zinc finger protein 438 | 2.27 | 1.2E-03 | up |
| *ACVR1B* | Activin A receptor type 1B | 2.25 | 6.9E-05 | up |
| *CEACAM4* | Carcinoembryonic antigen related cell adhesion molecule 4 | 2.23 | 8.1E-05 | up |
| *DUSP1* | Dual specificity phosphatase 1 | 2.22 | 9.8E-05 | up |
| *MAPK14* | Mitogen-activated protein kinase 14 | 2.21 | 4.7E-04 | up |
| *TPK1* | Thiamin pyrophosphokinase 1 | 2.20 | 8.8E-05 | up |
| *GPR27* | G protein-coupled receptor 27 | 2.15 | 5.7E-04 | up |
| *DYSF* | Dysferlin | 2.12 | 2.8E-03 | up |
| *CCDC71L* | Coiled-coil domain containing 71 like | 2.11 | 6.1E-04 | up |
| *ALOX5* | Arachidonate 5-lipoxygenase | 2.10 | 1.2E-03 | up |
| *WDFY3* | WD repeat and FYVE domain containing 3 | 2.08 | 1.2E-03 | up |
| *TLR8* | Toll like receptor 8 | 2.08 | 8.1E-04 | up |
| *HIF1A* | Hypoxia inducible factor 1 subunit alpha | 2.07 | 7.4E-04 | up |
| *TCTEX1D1* | Tctex1 domain containing 1 | 2.06 | 2.1E-03 | up |
| *PLIN5* | Perilipin 5 | 2.05 | 1.7E-03 | up |
| *PPP1R3D* | Protein phosphatase 1 regulatory subunit 3D | 2.05 | 2.6E-04 | up |
| *TMED8* | Transmembrane p24 trafficking protein family member 8 | 2.04 | 9.9E-06 | up |
| *LPL* | Lipoprotein lipase | 2.04 | 2.4E-03 | up |
| *PYGL* | Glycogen phosphorylase L | 2.01 | 2.9E-03 | up |
| *ITGAM* | Integrin subunit alpha M | 2.00 | 1.7E-03 | up |
| *CD160* | CD160 molecule | 9.42 | 2.5E-09 | down |
| *FCRL6* | Fc receptor like 6 | 8.35 | 4.9E-06 | down |
| *ADGRG1* | Adhesion G protein-coupled receptor G1 | 8.27 | 4.3E-06 | down |
| *GZMM* | Granzyme M | 5.90 | 4.6E-06 | down |
| *CLIC3* | Chloride intracellular channel 3 | 5.65 | 7.1E-06 | down |
| *XCL2* | X-C motif chemokine ligand 2 | 5.39 | 6.3E-07 | down |
| *TCL1A* | T cell leukemia/lymphoma 1A | 5.19 | 1.3E-05 | down |
| *GPR18* | G protein-coupled receptor 18 | 4.96 | 2.6E-07 | down |
| *GPR174* | G protein-coupled receptor 174 | 4.89 | 3.9E-06 | down |
| *CXCR5* | C-X-C motif chemokine receptor 5 | 4.80 | 1.3E-05 | down |
| *FCER2* | Fc fragment of IgE receptor II | 4.36 | 4.0E-05 | down |
| *LGALS2* | Galectin 2 | 4.34 | 5.3E-05 | down |
| *HLA-DPB1* | Major histocompatibility complex, class II, DP beta 1 | 4.32 | 5.3E-05 | down |
| *CD22* | CD22 molecule | 4.20 | 1.0E-04 | down |
| *PTGDR2* | Prostaglandin D2 receptor 2 | 4.10 | 8.4E-05 | down |
| *HLA-DOB* | Major histocompatibility complex, class II, DO beta | 3.93 | 3.9E-05 | down |
| *HLA-DOA* | Major histocompatibility complex, class II, DO alpha | 3.91 | 7.7E-06 | down |
| *PYHIN1* | Pyrin and HIN domain family member 1 | 3.85 | 6.2E-05 | down |
| *RASGRP1* | RAS guanyl releasing protein 1 | 3.85 | 6.8E-06 | down |
| *CD72* | CD72 molecule | 3.84 | 3.0E-06 | down |
| *NCR3* | Natural cytotoxicity triggering receptor 3 | 3.83 | 1.2E-05 | down |
| *MYBL1* | MYB proto-oncogene like 1 | 3.79 | 3.1E-05 | down |
| *MS4A1* | Membrane spanning 4-domains A1 | 3.72 | 2.3E-04 | down |
| *FLT3LG* | Fms related tyrosine kinase 3 ligand | 3.70 | 1.9E-06 | down |
| *VPREB3* | V-set pre-B cell surrogate light chain 3 | 3.56 | 2.5E-05 | down |
| *LPAR5* | Lysophosphatidic acid receptor 5 | 3.43 | 1.3E-05 | down |
| *PIK3C2B* | Phosphatidylinositol-4-phosphate 3-kinase catalytic subunit type 2 beta | 3.37 | 3.4E-05 | down |
| *SNX29P2* | Sorting nexin 29 pseudogene 2 | 3.37 | 1.4E-05 | down |
| *PAX5* | Paired box 5 | 3.33 | 1.6E-04 | down |
| *LBH* | LBH regulator of WNT signaling pathway | 3.31 | 1.7E-05 | down |
| *CYSLTR2* | Cysteinyl leukotriene receptor 2 | 3.30 | 4.7E-07 | down |
| *FCRLA* | Fc receptor like A | 3.25 | 1.4E-03 | down |
| *ZNF683* | Zinc finger protein 683 | 3.24 | 2.6E-05 | down |
| *CRIP2* | Cysteine rich protein 2 | 3.14 | 6.6E-05 | down |
| *ERBB2* | Erb-b2 receptor tyrosine kinase 2 | 3.13 | 1.5E-05 | down |
| *LDOC1* | LDOC1 regulator of NFKB signaling | 3.12 | 5.7E-05 | down |
| *TRABD2A* | TraB domain containing 2A | 3.11 | 9.6E-06 | down |
| *HABP4* | Hyaluronan binding protein 4 | 3.05 | 2.8E-06 | down |
| *NDRG2* | NDRG family member 2 | 3.02 | 1.7E-06 | down |
| *HLA-DRA* | Major histocompatibility complex, class II, DR alpha | 3.01 | 7.0E-04 | down |
| *BTLA* | B and T lymphocyte associated | 2.99 | 1.5E-04 | down |
| *PCBP4* | Poly(rC) binding protein 4 | 2.97 | 1.0E-04 | down |
| *CD101* | CD101 molecule | 2.97 | 4.3E-04 | down |
| *CROCC* | Ciliary rootlet coiled-coil, rootletin | 2.94 | 6.3E-06 | down |
| *ZNF483* | Zinc finger protein 483 | 2.91 | 6.8E-07 | down |
| *AK5* | Adenylate kinase 5 | 2.86 | 5.4E-06 | down |
| *CA5B* | Carbonic anhydrase 5B | 2.85 | 4.4E-05 | down |
| *CD79A* | CD79a molecule | 2.83 | 2.4E-03 | down |
| *CD200* | CD200 molecule | 2.81 | 2.9E-05 | down |
| *PVRIG* | PVR related immunoglobulin domain containing | 2.80 | 4.8E-06 | down |
| *CYP4V2* | Cytochrome P450 family 4 subfamily V member 2 | 2.79 | 1.9E-04 | down |
| *CHI3L2* | Chitinase 3 like 2 | 2.77 | 8.3E-04 | down |
| *BLK* | BLK proto-oncogene, Src family tyrosine kinase | 2.77 | 1.3E-03 | down |
| *MLLT3* | MLLT3 super elongation complex subunit | 2.75 | 7.4E-06 | down |
| *APBA2* | Amyloid beta precursor protein binding family A member 2 | 2.74 | 2.8E-05 | down |
| *FBXL16* | F-box and leucine rich repeat protein 16 | 2.72 | 4.4E-05 | down |
| *TMEM229B* | Transmembrane protein 229B | 2.70 | 4.5E-04 | down |
| *LAT* | Linker for activation of T cells | 2.69 | 6.4E-06 | down |
| *NMUR1* | Neuromedin U receptor 1 | 2.68 | 4.7E-05 | down |
| *CASS4* | Cas scaffold protein family member 4 | 2.68 | 2.5E-03 | down |
| *SFMBT2* | Scm like with four mbt domains 2 | 2.67 | 2.1E-04 | down |
| *AGMAT* | Agmatinase | 2.67 | 4.8E-05 | down |
| *ZXDB* | Zinc finger X-linked duplicated B | 2.66 | 4.1E-06 | down |
| *GPR68* | G protein-coupled receptor 68 | 2.66 | 4.4E-06 | down |
| *HIVEP3* | HIVEP zinc finger 3 | 2.65 | 3.5E-04 | down |
| *RHOF* | Ras homolog family member F, filopodia associated | 2.65 | 9.8E-06 | down |
| *ATP1A3* | ATPase Na+/K+ transporting subunit alpha 3 | 2.64 | 9.0E-06 | down |
| *ADRB2* | Adrenoceptor beta 2 | 2.64 | 6.2E-05 | down |
| *DOCK10* | Dedicator of cytokinesis 10 | 2.63 | 8.0E-05 | down |
| *KLHL3* | Kelch like family member 3 | 2.63 | 9.1E-09 | down |
| *CCN3* | Cellular communication network factor 3 | 2.63 | 3.6E-04 | down |
| *APOL3* | Apolipoprotein L3 | 2.63 | 1.6E-03 | down |
| *PLEKHO1* | Pleckstrin homology domain containing O1 | 2.63 | 1.4E-05 | down |
| *MAN1C1* | Mannosidase alpha class 1C member 1 | 2.59 | 7.2E-08 | down |
| *RHOBTB2* | Rho related BTB domain containing 2 | 2.59 | 1.3E-04 | down |
| *LTA* | Lymphotoxin alpha | 2.58 | 1.5E-04 | down |
| *USP28* | Ubiquitin specific peptidase 28 | 2.58 | 7.7E-05 | down |
| *CCDC88C* | Coiled-coil domain containing 88C | 2.58 | 7.3E-05 | down |
| *LDLRAD4* | Low density lipoprotein receptor class A domain containing 4 | 2.56 | 2.0E-05 | down |
| *ZDHHC14* | Zinc finger DHHC-type containing 14 | 2.56 | 2.9E-05 | down |
| *UTP20* | UTP20 small subunit processome component | 2.55 | 4.2E-04 | down |
| *NOL6* | Nucleolar protein 6 | 2.55 | 4.4E-04 | down |
| *DNPEP* | Aspartyl aminopeptidase | 2.53 | 1.6E-04 | down |
| *ZXDA* | Zinc finger X-linked duplicated A | 2.53 | 3.2E-07 | down |
| *GSE1* | Gse1 coiled-coil protein | 2.51 | 5.7E-05 | down |
| *MRPL4* | Mitochondrial ribosomal protein L4 | 2.51 | 9.3E-04 | down |
| *EFNB1* | Ephrin B1 | 2.50 | 1.4E-04 | down |
| *EXOG* | Exo/endonuclease G | 2.50 | 1.2E-04 | down |
| *CEP290* | Centrosomal protein 290 | 2.48 | 1.1E-05 | down |
| *ZFPM1* | Zinc finger protein, FOG family member 1 | 2.48 | 2.9E-04 | down |
| *RPS6KA5* | Ribosomal protein S6 kinase A5 | 2.45 | 6.0E-05 | down |
| *ARRB1* | Arrestin beta 1 | 2.44 | 1.4E-05 | down |
| *OBSCN* | Obscurin, cytoskeletal calmodulin and titin-interacting RhoGEF | 2.43 | 1.4E-04 | down |
| *PPP1R13B* | Protein phosphatase 1 regulatory subunit 13B | 2.43 | 5.2E-05 | down |
| *CTSO* | Cathepsin O | 2.42 | 4.3E-05 | down |
| *TMEM263* | Transmembrane protein 263 | 2.42 | 4.2E-04 | down |
| *S1PR5* | Sphingosine-1-phosphate receptor 5 | 2.42 | 1.5E-03 | down |
| *LINC00926* | Long intergenic non-protein coding RNA 926 | 2.42 | 1.1E-03 | down |
| *NIPA1* | NIPA magnesium transporter 1 | 2.40 | 2.6E-06 | down |
| *GPR162* | G protein-coupled receptor 162 | 2.39 | 5.5E-05 | down |
| *NOP14* | NOP14 nucleolar protein | 2.39 | 6.1E-05 | down |
| *VCL* | Vinculin | 2.39 | 2.0E-03 | down |
| *SMYD2* | SET and MYND domain containing 2 | 2.38 | 6.9E-06 | down |
| *RRP7A* | Ribosomal RNA processing 7 homolog A | 2.38 | 7.3E-04 | down |
| *PRKX* | Protein kinase X-linked | 2.37 | 3.0E-04 | down |
| *CHIC1* | Cysteine rich hydrophobic domain 1 | 2.37 | 5.4E-05 | down |
| *SH2D3A* | SH2 domain containing 3A | 2.37 | 4.9E-04 | down |
| *SNURF* | SNRPN upstream reading frame | 2.36 | 2.0E-05 | down |
| *LTB* | Lymphotoxin beta | 2.35 | 2.2E-05 | down |
| *ZNF548* | Zinc finger protein 548 | 2.33 | 1.4E-05 | down |
| *POGLUT3* | Protein O-glucosyltransferase 3 | 2.33 | 8.2E-05 | down |
| *ZNF853* | Zinc finger protein 853 | 2.32 | 7.2E-05 | down |
| *CACNA2D2* | Calcium voltage-gated channel auxiliary subunit alpha2delta 2 | 2.31 | 4.2E-04 | down |
| *SNPH* | Syntaphilin | 2.31 | 9.4E-05 | down |
| *PKIA* | cAMP-dependent protein kinase inhibitor alpha | 2.31 | 1.4E-04 | down |
| *TPPP3* | Tubulin polymerization promoting protein family member 3 | 2.30 | 2.3E-03 | down |
| *NOM1* | Nucleolar protein with MIF4G domain 1 | 2.30 | 6.3E-04 | down |
| *SLC9A7* | Solute carrier family 9 member A7 | 2.29 | 1.1E-04 | down |
| *PATZ1* | POZ/BTB and AT hook containing zinc finger 1 | 2.29 | 2.5E-05 | down |
| *REXO4* | REX4 homolog, 3'-5' exonuclease | 2.28 | 6.7E-05 | down |
| *PRSS23* | Serine protease 23 | 2.28 | 2.1E-04 | down |
| *SLC4A4* | Solute carrier family 4 member 4 | 2.28 | 6.2E-05 | down |
| *CEP126* | Centrosomal protein 126 | 2.27 | 2.3E-06 | down |
| *RPUSD2* | RNA pseudouridine synthase domain containing 2 | 2.27 | 6.3E-04 | down |
| *PIK3R6* | Phosphoinositide-3-kinase regulatory subunit 6 | 2.27 | 8.8E-07 | down |
| *MSANTD2* | Myb/SANT DNA binding domain containing 2 | 2.27 | 5.1E-05 | down |
| *TPCN1* | Two pore segment channel 1 | 2.27 | 5.6E-05 | down |
| *ZNF571* | Zinc finger protein 571 | 2.27 | 1.9E-06 | down |
| *CCR4* | C-C motif chemokine receptor 4 | 2.26 | 4.8E-04 | down |
| *PABPC3* | Poly(A) binding protein cytoplasmic 3 | 2.25 | 1.0E-04 | down |
| *PEA15* | Proliferation and apoptosis adaptor protein 15 | 2.25 | 5.5E-04 | down |
| *ICOSLG* | Inducible T cell costimulator ligand | 2.24 | 1.0E-03 | down |
| *LOC389906* | Zinc finger protein 839 pseudogene | 2.24 | 1.2E-04 | down |
| *CFAP36* | Cilia and flagella associated protein 36 | 2.24 | 1.3E-05 | down |
| *EARS2* | Glutamyl-tRNA synthetase 2, mitochondrial | 2.23 | 3.0E-04 | down |
| *EPHA4* | EPH receptor A4 | 2.22 | 3.6E-04 | down |
| *IGFBP3* | Insulin like growth factor binding protein 3 | 2.22 | 3.4E-04 | down |
| *IL11RA* | Interleukin 11 receptor subunit alpha | 2.21 | 2.7E-05 | down |
| *LMTK3* | Lemur tyrosine kinase 3 | 2.20 | 3.0E-04 | down |
| *ICAM2* | Intercellular adhesion molecule 2 | 2.20 | 1.6E-04 | down |
| *LINC00299* | Long intergenic non-protein coding RNA 299 | 2.20 | 2.1E-03 | down |
| *NARS2* | Asparaginyl-tRNA synthetase 2, mitochondrial | 2.19 | 1.3E-03 | down |
| *ZC3H8* | Zinc finger CCCH-type containing 8 | 2.17 | 9.6E-06 | down |
| *ARHGEF19* | Rho guanine nucleotide exchange factor 19 | 2.17 | 4.1E-05 | down |
| *KIF5C* | Kinesin family member 5C | 2.17 | 5.1E-04 | down |
| *GPA33* | Glycoprotein A33 | 2.17 | 2.8E-04 | down |
| *LOC100505549* | Uncharacterized LOC100505549 | 2.17 | 3.9E-04 | down |
| *CCDC102A* | Coiled-coil domain containing 102A | 2.17 | 6.6E-05 | down |
| *FAM227B* | Family with sequence similarity 227 member B | 2.16 | 1.4E-04 | down |
| *SETD6* | SET domain containing 6, protein lysine methyltransferase | 2.15 | 5.5E-05 | down |
| *ZNF573* | Zinc finger protein 573 | 2.15 | 2.4E-05 | down |
| *GALNT12* | Polypeptide N-acetylgalactosaminyltransferase 12 | 2.15 | 1.1E-05 | down |
| *RANGAP1* | Ran GTPase activating protein 1 | 2.15 | 7.3E-04 | down |
| *PTER* | Phosphotriesterase related | 2.14 | 3.8E-04 | down |
| *L3MBTL2* | L3MBTL histone methyl-lysine binding protein 2 | 2.14 | 9.6E-04 | down |
| *KIAA1328* | KIAA1328 | 2.14 | 1.8E-04 | down |
| *STK39* | Serine/threonine kinase 39 | 2.13 | 2.9E-05 | down |
| *GFI1B* | Growth factor independent 1B transcriptional repressor | 2.13 | 8.8E-04 | down |
| *FAM120C* | Family with sequence similarity 120C | 2.13 | 2.5E-05 | down |
| *LAS1L* | LAS1 like, ribosome biogenesis factor | 2.13 | 2.0E-03 | down |
| *GSPT2* | G1 to S phase transition 2 | 2.13 | 2.8E-05 | down |
| *ZNF485* | Zinc finger protein 485 | 2.13 | 3.2E-06 | down |
| *ITGA6* | Integrin subunit alpha 6 | 2.12 | 3.6E-05 | down |
| *FAM50B* | Family with sequence similarity 50 member B | 2.12 | 2.5E-04 | down |
| *SMPD3* | Sphingomyelin phosphodiesterase 3 | 2.12 | 1.7E-04 | down |
| *PDZD4* | PDZ domain containing 4 | 2.12 | 4.5E-04 | down |
| *TCEAL3* | Transcription elongation factor A like 3 | 2.12 | 4.2E-04 | down |
| *CAMKMT* | Calmodulin-lysine N-methyltransferase | 2.12 | 1.9E-05 | down |
| *TRMT10B* | tRNA methyltransferase 10B | 2.12 | 5.3E-05 | down |
| *MDC1* | Mediator of DNA damage checkpoint 1 | 2.12 | 1.4E-03 | down |
| *ADGRL1* | Adhesion G protein-coupled receptor L1 | 2.12 | 8.0E-05 | down |
| *SGPP1* | Sphingosine-1-phosphate phosphatase 1 | 2.11 | 2.0E-04 | down |
| *MAK16* | MAK16 homolog | 2.11 | 2.3E-04 | down |
| *RPS27* | Ribosomal protein S27 | 2.11 | 5.7E-05 | down |
| *PDLIM2* | PDZ and LIM domain 2 | 2.11 | 3.7E-06 | down |
| *KMT2A* | Lysine methyltransferase 2A | 2.11 | 4.4E-05 | down |
| *UBE2Q2* | Ubiquitin conjugating enzyme E2 Q2 | 2.10 | 6.1E-04 | down |
| *POU6F1* | POU class 6 homeobox 1 | 2.10 | 2.4E-04 | down |
| *TRANK1* | Tetratricopeptide repeat and ankyrin repeat containing 1 | 2.10 | 2.4E-04 | down |
| *GIMAP6* | GTPase, IMAP family member 6 | 2.10 | 8.3E-04 | down |
| *BEX2* | Brain expressed X-linked 2 | 2.10 | 1.2E-04 | down |
| *DDX24* | DEAD-box helicase 24 | 2.09 | 3.5E-04 | down |
| *KNOP1* | Lysine rich nucleolar protein 1 | 2.09 | 1.4E-04 | down |
| *UNK* | Unk zinc finger | 2.09 | 1.9E-05 | down |
| *PARP16* | Poly(ADP-ribose) polymerase family member 16 | 2.09 | 2.0E-04 | down |
| *FAM53B* | Family with sequence similarity 53 member B | 2.08 | 9.0E-04 | down |
| *CMC1* | C-X9-C motif containing 1 | 2.08 | 7.5E-05 | down |
| *TTC12* | Tetratricopeptide repeat domain 12 | 2.08 | 4.6E-05 | down |
| *ZNF527* | Zinc finger protein 527 | 2.07 | 3.2E-05 | down |
| *NLE1* | Notchless homolog 1 | 2.07 | 9.9E-04 | down |
| *DENND2D* | DENN domain containing 2D | 2.07 | 1.3E-05 | down |
| *CCDC92* | Coiled-coil domain containing 92 | 2.07 | 1.7E-04 | down |
| *PAIP2B* | Poly(A) binding protein interacting protein 2B | 2.07 | 2.4E-05 | down |
| *PAXX* | PAXX non-homologous end joining factor | 2.07 | 2.0E-04 | down |
| *NLRP1* | NLR family pyrin domain containing 1 | 2.06 | 1.6E-04 | down |
| *GNAO1* | G protein subunit alpha o1 | 2.05 | 2.7E-03 | down |
| *ZNF354C* | Zinc finger protein 354C | 2.05 | 3.3E-04 | down |
| *DYRK2* | Dual specificity tyrosine phosphorylation regulated kinase 2 | 2.05 | 3.4E-04 | down |
| *SLC25A26* | Solute carrier family 25 member 26 | 2.05 | 7.2E-04 | down |
| *PDGFD* | Platelet derived growth factor D | 2.05 | 4.0E-04 | down |
| *PIGM* | Phosphatidylinositol glycan anchor biosynthesis class M | 2.04 | 3.3E-03 | down |
| *USP46* | Ubiquitin specific peptidase 46 | 2.04 | 4.3E-04 | down |
| *TRIM44* | Tripartite motif containing 44 | 2.03 | 4.0E-04 | down |
| *HEATR1* | HEAT repeat containing 1 | 2.03 | 7.8E-04 | down |
| *IPO4* | Importin 4 | 2.03 | 2.0E-04 | down |
| *SOGA1* | Suppressor of glucose, autophagy associated 1 | 2.02 | 9.1E-05 | down |
| *MFSD6* | Major facilitator superfamily domain containing 6 | 2.02 | 3.7E-04 | down |
| *CCDC28B* | Coiled-coil domain containing 28B | 2.02 | 1.4E-04 | down |
| *KLHL42* | Kelch like family member 42 | 2.02 | 1.4E-04 | down |
| *THTPA* | Thiamine triphosphatase | 2.02 | 2.3E-04 | down |
| *AKT3* | AKT serine/threonine kinase 3 | 2.02 | 8.9E-06 | down |
| *TMEM99* | Transmembrane protein 99 (putative) | 2.01 | 1.4E-04 | down |
| *HHLA3* | HERV-H LTR-associating 3 | 2.01 | 2.7E-04 | down |
| *RPL32* | Ribosomal protein L32 | 2.01 | 3.5E-04 | down |
| *SARS2* | Seryl-tRNA synthetase 2, mitochondrial | 2.00 | 1.7E-03 | down |
| *LCK* | LCK proto-oncogene, Src family tyrosine kinase | 2.00 | 1.6E-07 | down |
| *CUL1* | Cullin 1 | 2.00 | 1.9E-03 | down |
| *TMEM42* | Transmembrane protein 42 | 2.00 | 9.5E-05 | down |

**Supplementary Table 3.** Enriched functional analysis of 65 up-regulated and 218 down-regulated genes in non-survivors. The biological functions were analysed using MetaScape.

| **Up-regulation** |  |  |  |
| --- | --- | --- | --- |
| **Term** | **Accession** | **No. of genes** | **Gene** |
| GO:0002274 | Myeloid leukocyte activation | 14 | *ALOX5, MAPK14, ITGAM, PYGL, S100A9, SLC2A3, TLR2, TLR4, DYSF, ADAM9, IL18RAP, MGAM, TLR8, CLEC4D* |
| R-HSA-168898 | Toll-like Receptor Cascades | 8 | *MAPK14, ITGAM, NFKBIA, S100A9, TLR2, TLR4, IRAK3, TLR8* |
| GO:0072593 | Reactive oxygen species metabolic process | 8 | *MAPK14, GADD45A, HIF1A, ITGAM, TLR2, TLR4, SH3PXD2B, PLIN5* |
| GO:0045648 | Positive regulation of erythrocyte differentiation | 4 | *ACVR1B, MAPK14, HIF1A, HMGB2* |
| GO:0038066 | p38MAPK cascade | 4 | *MAPK14, GADD45A, DUSP1, PER1* |
| GO:0045600 | Positive regulation of fat cell differentiation | 4 | *MAPK14, LPL, CCDC71L, SH3PXD2B* |
| GO:0019915 | Lipid storage | 4 | *LPL, NFKBIA, DYSF, PLIN5* |
| R-HSA-449147 | Signaling by Interleukins | 8 | *ALOX5, MAPK14, HIF1A, ITGAM, NFKBIA, IL1R2, IL18RAP, IRAK3* |
| GO:0009251 | Glucan catabolic process | 3 | *PPP1R3D, PYGL, MGAM* |
| GO:0005975 | Carbohydrate metabolic process | 9 | *MAPK14, HIF1A, PFKFB3, PPP1R3D, PYGL, SLC2A3, DYSF, MGAM, KL* |
| GO:0006909 | Phagocytosis | 7 | *CEACAM4, ITGAM, MYO10, TLR2, TLR4, DYSF, MERTK* |
| M12705 | SIG CD40PATHWAYMAP | 3 | *MAPK14, DUSP1, NFKBIA* |
| GO:0009746 | Response to hexose | 5 | *GPR27, HIF1A, LPL, KLF7, SLC26A6* |
| GO:0007140 | Male meiotic nuclear division | 3 | *CCNA1, SYCP2, TDRD9* |
| R-HSA-6791312 | TP53 regulating transcription of cell cycle genes | 3 | *PLK3, GADD45A, CCNA1* |
| GO:0031331 | Positive regulation of cellular catabolic process | 6 | *PLK3, HIF1A, PFKFB3, ADAM9, RNF144B, PLIN5* |
| GO:0022411 | Cellular component disassembly | 7 | *ASPH,PLK3, HIF1A, HMGB2, ITGAM, IRAK3, SH3PXD2B* |
| GO:0006979 | Response to oxidative stress | 6 | *PLK3, DUSP1, HIF1A, TLR4, ADAM9, IL18RAP* |
| GO:0043470 | Regulation of carbohydrate catabolic process | 3 | *HIF1A, PFKFB3, PPP1R3D* |
| GO:0032787 | Monocarboxylic acid metabolic process | 7 | *ALOX5, MAPK14, HIF1A, LPL, PFKFB3, PECR, PLIN5* |
|  |  |  |  |
| **Down regulation** |  |  |  |
| **Term** | **Accession** | **No. of genes** | **Gene** |
| hsa04640 | Hematopoietic cell lineage | 10 | *MS4A1, CD22, FCER2, FLT3LG, HLA-DOA, HLA-DOB, HLA-DPB1, HLA-DRA, IL11RA, ITGA6* |
| R-HSA-1280218 | Adaptive Immune System | 24 | *BLK, CD22, CD79A, CTSO, HLA-DOA, HLA-DOB, HLA-DPB1, HLA-DRA, ICAM2, LCK,CD200,CUL1,CD101,AKT3,RASGRP1,CD160,ICOSLG,KLHL3,LAT,KLHL42,UBE2Q2,FBXL16,BTLA,NCR3* |
| GO:0046649 | Lymphocyte activation | 23 | *CXCR5, MS4A1, CD22, CD79A, EFNB1, ERBB2, FLT3LG, GPR18, HLA-DOA, HLA-DPB1, LCK, RASGRP1, CD160, ICOSLG, PATZ1, LAT, DOCK10, ZC3H8, PIK3R6, BTLA, ZFPM1, ZNF683, NCR3* |
| GO:0030098 | Lymphocyte differentiation | 14 | *MS4A1, CD79A, ERBB2, FLT3LG, GPR18, HLA-DOA, LCK, RASGRP1, PATZ1, DOCK10, ZC3H8, PIK3R6, ZFPM1, ZNF683* |
| GO:0042274 | Ribosomal small subunit biogenesis | 6 | *RPS27, NOP14, UTP20, RRP7A, HEATR1, NOM1* |
| GO:0048872 | Homeostasis of number of cells | 10 | *CCR4, FLT3LG, CCN3, AKT3, LAT, NLE1, DOCK10, ZC3H8, GPR174, ZFPM1* |
| GO:0035025 | Positive regulation of Rho protein signal transduction | 4 | *ARRB1, GPR18, ADGRG1, GPR174* |
| R-HSA-373076 | Class A/1 (Rhodopsin-like receptors) | 11 | *ADRB2, CXCR5, CCR4, GPR18, XCL2, GPR68, NMUR1, PTGDR2, S1PR5, CYSLTR2, LPAR5* |
| M177 | PID EPHA FWDPATHWAY | 4 | *BLK, EPHA4, LCK, PIK3R6* |
| GO:0071902 | Positive regulation of protein serine/threonine kinase activity | 11 | *ADRB2, ARRB1, EPHA4, ERBB2, TCL1A, PEA15, RASGRP1, STK39, PARP16, PDGFD, PIK3R6* |
| GO:0032729 | Positive regulation of interferon-gamma production | 5 | *HLA-DPB1, LTA, RASGRP1, CD160, ZFPM1* |
| M34 | PID TCR PATHWAY | 5 | *HLA-DRA, LCK, RASGRP1, LAT, STK39* |
| GO:0048535 | Lymph node development | 3 | *CXCR5, LTA, LTB* |
| GO:0032793 | Positive regulation of CREB transcription factor activity | 3 | *CD200, RPS6KA5, LPAR5* |
| R-HSA-379726 | Mitochondrial tRNA aminoacylation | 3 | *SARS2, NARS2, EARS2* |
| M155 | PID S1P META PATHWAY | 3 | *GNAO1, S1PR5, SGPP1* |
| GO:0035162 | Embryonic hemopoiesis | 3 | *FLT3LG, KMT2A, ZFPM1* |
| GO:0050853 | B cell receptor signaling pathway | 6 | *BLK, MS4A1, CD22, CD79A, LCK, PAX5* |
| hsa04261 | Adrenergic signaling in cardiomyocytes | 6 | *ADRB2, ATP1A3, RPS6KA5, CACNA2D2, AKT3, PIK3R6* |
| GO:0008045 | Motor neuron axon guidance | 3 | *EPHA4, ERBB2, KIF5C* |

**Supplementary Table 4.** Summary of KEGG pathways of up-regulated and down-regulated genes in whole blood of non-survivors compared with survivors.

| **Regulation** | **Term** | **KEGG pathway** | **Log10 (P)** | **Log10 (Q)** | **Number of gene** | **Genes symbols** |
| --- | --- | --- | --- | --- | --- | --- |
| **Up** | hsa04620 | Toll-like receptor signaling pathway | -5.08 | -2.55 | 5/104 | *MAPK14, NFKBIA,*  *TLR2, TLR4, TLR8* |
|  | hsa04659 | Th17 cell differentiation | -2.54 | -0.62 | 3/107 | *MAPK14, HIF1A,*  *NFKBIA* |
|  | hsa04010 | MAPK signaling pathway | -2.33 | -0.46 | 4/255 | *MAPK14, GADD45A,*  *DUSP1, IL1R2* |
|  | hsa04657 | IL-17 signaling pathway | -2.71 | -0.74 | 3/93 | *MAPK14, NFKBIA,*  *S100A9* |
|  | hsa04068 | FoxO signaling pathway | -2.28 | -0.42 | 3/132 | *PLK3, MAPK14,*  *GADD45A* |
|  | hsa04066 | HIF-1 signaling pathway | -2.61 | -0.67 | 3/101 | *HIF1A, PFKFB3,*  *TLR4* |
|  |  |  |  |  |  |  |
| **Down** | hsa04640 | Hematopoietic cell lineage | -7.73 | -3.42 | 10/97 | *MS4A1, CD22,*  *FCER2, FLT3LG,*  *HLA-DOA, HLA-DOB, HLA-DPB1, HLA-DRA,*  *IL11RA, ITGA6* |
|  | hsa04514 | Cell adhesion molecules (CAMs) | -4.27 | -1.19 | 8/145 | *CD22, HLA-DOA,*  *HLA-DOB, HLA-DPB1,*  *HLA-DRA, ICAM2,*  *ITGA6, ICOSLG* |
|  | hsa04672 | Intestinal immune network for IgA production | -4.10 | -1.11 | 5/49 | *HLA-DOA, HLA-DOB, HLA-DPB1, HLA-DRA, ICOSLG* |
|  | hsa04658 | Th1 and Th2 cell differentiation | -3.72 | -0.92 | 6/92 | *HLA-DOA, HLA-DOB, HLA-DPB1, HLA-DRA, LCK, LAT* |
|  | hsa04659 | Th17 cell differentiation | -3.37 | -0.73 | 6/107 | *HLA-DOA, HLA-DOB, HLA-DPB1, HLA-DRA, LCK, LAT* |
|  | hsa04612 | Antigen processing and presentation | -2.28 | -0.09 | 4/77 | *HLA-DOA, HLA-DOB, HLA-DPB1, HLA-DRA* |
|  | hsa04662 | B cell receptor signaling pathway | -2.41 | -0.19 | 4/71 | *CD22, CD72,*  *CD79A, AKT3* |

P, *P* value; Q, *P* value adjusted using the Benjamini-Hochberg procedure; hsa, *Homo sapient*

**Supplementary Table 5.** *P* values of Dunn's multiple comparisons test of differentially expressed genes in whole blood among groups of non-survivors, survivors, and healthy controls.

| **Gene ID** | **Regulation** | ***P* value** | | |
| --- | --- | --- | --- | --- |
|  |  | **NS *vs* S** | **NS *vs* HC** | **S *vs* HC** |
| *IL1R2* | Up | < 0.0001 | < 0.0001 | 0.0032 |
| *HMGB2* | Up | < 0.0001 | 0.0001 | > 0.9999 |
| *GADD45A* | Up | 0.0002 | < 0.0001 | 0.1096 |
| *TLR4* | Up | 0.0004 | < 0.0001 | 0.0004 |
| *GAS7* | Up | 0.0004 | < 0.0001 | 0.0105 |
| *S100A9* | Up | 0.0005 | < 0.0001 | < 0.0001 |
| *GPR27* | Up | 0.0009 | < 0.0001 | 0.0018 |
| *IL18RAP* | Up | 0.0013 | < 0.0001 | 0.0033 |
| *MGAM* | Up | 0.0015 | < 0.0001 | 0.0002 |
| *HIF1A* | Up | 0.0020 | < 0.0001 | 0.0091 |
| *IRAK3* | Up | 0.0023 | < 0.0001 | < 0.0001 |
| *MAPK14* | Up | 0.0038 | < 0.0001 | < 0.0001 |
| *NFKBIA* | Up | 0.0040 | < 0.0001 | < 0.0001 |
| *TLR2* | Up | 0.0047 | < 0.0001 | 0.0007 |
| *ITGAM* | Up | 0.0054 | < 0.0001 | 0.0017 |
| *PLK3* | Up | 0.0100 | < 0.0001 | 0.0010 |
| *FKBP5* | Up | 0.0110 | < 0.0001 | 0.0002 |
| *CD160* | Down | 0.0159 | < 0.0001 | < 0.0001 |
| *PER1* | Up | 0.0383 | < 0.0001 | < 0.0001 |
| *HLA-DPB1* | Down | 0.0579 | > 0.9999 | 0.0966 |
| *HLA-DOA* | Down | 0.1274 | > 0.9999 | 0.7229 |
| *CD22* | Down | 0.1986 | 0.6618 | 0.0239 |
| *GPR56* | Down | 0.3047 | > 0.9999 | 0.3343 |
| *LPL* | Up | 0.5096 | < 0.0001 | < 0.0001 |
| *ACVR1B* | Up | 0.4826 | < 0.0001 | < 0.0001 |
| *LCK* | Down | 0.6225 | 0.2826 | 0.0222 |
| *CD72* | Down | 0.6719 | > 0.9999 | 0.3161 |
| *LAT* | Down | 0.8859 | 0.0793 | 0.0093 |

NS, non-survivors; S, survivors; HC, healthy controls

**Supplementary Table 6.** Area under the receiver operating characteristic curves (AUROCC) of 28 DEGs in discrimination between non-survivors and survivors.

| **Gene ID** | **Regulation** | **AUROCC (95% CI)** | | | | | |
| --- | --- | --- | --- | --- | --- | --- | --- |
|  |  | **NS *vs* S** | | **NS *vs* HC** | | **S *vs* HC** | |
| *S100A9* | Up | 0.88 | (0.79-0.97) | 1.00 | (1.00-1.00) | 1.00 | (1.00-1.00) |
| *IL1R2* | Up | 0.87 | (0.78-0.96) | 1.00 | (1.00-1.00) | 0.86 | (0.76-0.96) |
| *TLR4* | Up | 0.86 | (0.77-0.95) | 1.00 | (1.00-1.00) | 0.93 | (0.84-1.01) |
| *FKBP5* | Up | 0.85 | (0.74-0.96) | 1.00 | (1.00-1.00) | 1.00 | (1.00-1.00) |
| *IRAK3* | Up | 0.83 | (0.73-0.94) | 1.00 | (1.00-1.00) | 0.99 | (0.97-1.01) |
| *MGAM* | Up | 0.83 | (0.73-0.93) | 1.00 | (1.00-1.00) | 0.99 | (0.96-1.01) |
| *HMGB2* | Up | 0.82 | (0.71-0.94) | 0.87 | (0.77-0.97) | 0.51 | (0.34-0.67) |
| *MAPK14* | Up | 0.82 | (0.72-0.93) | 1.00 | (1.00-1.00) | 1.00 | (0.99-1.00) |
| *NFKBIA* | Up | 0.82 | (0.72-0.93) | 1.00 | (1.00-1.00) | 1.00 | (1.00-1.00) |
| *GAS7* | Up | 0.82 | (0.71-0.93) | 1.00 | (1.00-1.00) | 1.00 | (1.00-1.00) |
| *GADD45A* | Up | 0.82 | (0.70-0.93) | 1.00 | (0.95-1.02) | 0.82 | (0.69-0.96) |
| *GPR27* | Up | 0.82 | (0.71-0.93) | 1.00 | (1.00-1.00) | 1.00 | (1.00-1.00) |
| *IL18RAP* | Up | 0.80 | (0.68-0.91) | 0.99 | (0.98-1.01) | 0.83 | (0.72-0.94) |
| *CD160* | Down | 0.77 | (0.65-0.89) | 0.99 | (0.98-1.01) | 0.98 | (0.93-0.02) |
| *ITGAM* | Up | 0.77 | (0.65-0.89) | 1.00 | (0.98-1.01) | 1.00 | (1.00-1.00) |
| *TLR2* | Up | 0.77 | (0.65-0.89) | 1.00 | (1.00-1.00) | 0.85 | (0.75-0.96) |
| *HIF1A* | Up | 0.76 | (0.64-0.88) | 0.99 | (0.98-1.01) | 0.76 | (0.63-0.89) |
| *PLK3* | Up | 0.76 | (0.63-0.89) | 0.98 | (0.95-1.02) | 1.00 | (1.00-1.00) |
| *PER1* | Up | 0.75 | (0.63-0.88) | 1.00 | (1.00-1.00) | 1.00 | (1.00-1.00) |
| *HLA-DPB1* | Down | 0.68 | (0.54-0.82) | 0.51 | (0.32-0.70) | 0.68 | (0.53-0.83) |
| *HLA-DOA* | Down | 0.66 | (0.51-0.80) | 0.53 | (0.31-0.74) | 0.61 | (0.41-0.80) |
| *LPL* | Up | 0.64 | (0.50-0.78) | 1.00 | (1.00-1.00) | 1.00 | (1.00-1.00) |
| *ACVR1B* | Up | 0.64 | (0.49-0.78) | 1.00 | (1.00-1.00) | 1.00 | (1.00-1.00) |
| *CD22* | Down | 0.64 | (0.49-0.78) | 0.62 | (0.43-0.80) | 0.76 | (0.62-0.90) |
| *GPR56* | Down | 0.62 | (0.47-0.77) | 0.52 | (0.34-0.70) | 0.67 | (0.49-0.85) |
| *LCK* | Down | 0.59 | (0.44-0.74) | 0.65 | (0.48-0.81) | 0.76 | (0.62-0.90) |
| *CD72* | Down | 0.59 | (0.44-0.73) | 0.58 | (0.38-0.78) | 0.69 | (0.52-0.86) |
| *LAT* | Down | 0.58 | (0.44-0.73) | 0.74 | (0.59-0.90) | 0.81 | (0.67-0.94) |

Note: NS = Non-survivors, S = Survivors, and HC = Healthy controls.

**Supplementary Table 7.** *P* values of Mann-Whitney test of differentially expressed genes in melioidosis patients at different time points.

| **Gene ID** | **Median (IQR)** | | | | ***P* values** | | | | | | |  |
| --- | --- | --- | --- | --- | --- | --- | --- | --- | --- | --- | --- | --- |
|  | **Day 0** | **Day 5** | **Day 12** | **Day 28** | | **Day 0**  ***vs***  **Day 5** | **Day 0**  ***vs***  **Day 12** | **Day 0**  **vs**  **Day 28** | **Day 5**  **vs**  **Day 12** | **Day 5**  ***vs***  **Day 28** | **Day 12**  ***vs***  **Day 28** | |
| ***GAS7*** | 0.25  (0.06- 0.52) | 0.16  (0.04-0.41) | 0.04  (0.02-0.10) | 0.07  (0.03-0.11) | | 0.70 | 0.13 | 0.08 | 0.05 | 0.10 | 0.43 | |
| ***NFKBIA*** | 1.94  (0.40-2.78) | 0.92  (0.46-1.24) | 0.51  (0.32-0.55) | 0.25  (0.18-0.41) | | 0.23 | 0.13 | 0.08 | 0.08 | < 0.01 | 0.05 | |
| ***IL1R2*** | 1.52  (0.32-2.59) | 1.68  (0.39-2.88) | 0.54  (0.19-0.91) | 0.30  (0.21-0.48) | | 0.85 | 0.13 | 0.04 | 0.07 | 0.01 | 0.43 | |
| ***S100A9*** | 582.2  (144.30-927.20) | 229.3  (103.80-393.10) | 134.4  (59.45-191.80) | 74.68  (66.92-99.89) | | 0.23 | 0.02 | < 0.01 | 0.10 | 0.01 | 0.32 | |
| ***IRAK3*** | 0.44  (0.21-0.71) | 0.28  (0.18-0.76) | 0.10  (0.07-0.26) | 0.10  (0.08-0.12) | | 0.78 | < 0.01 | < 0.001 | 0.05 | 0.01 | 0.78 | |

**Supplementary Table 8. Temporal changes in gene expression of melioidosis patients relative to day 0, day 5, and day 12.**

| **Gene ID** | **Gene expression fold change of 8 individual patients**  **Day 5/Day 0** | | | | | | | | **Mean fold change**  **(95% CI)** |
| --- | --- | --- | --- | --- | --- | --- | --- | --- | --- |
|  | **50-076** | **50-080** | **50-081** | **50-091** | **50-092** | **50-208** | **50-209** | **50-211** |  |
| ***GAS7*** | 19.84 | 1.28 | 0.45 | 0.11 | 0.21 | 0.31 | 1.02 | 1.92 | 3.14  (-1.56 -7.84) |
| ***NFKBIA*** | 5.71 | 2.13 | 0.28 | 0.16 | 0.43 | 0.39 | 0.56 | 1.88 | 1.44  (0.14-2.74) |
| ***IL1R2*** | 1.10 | 0.66 | 0.71 | 0.68 | 0.64 | 0.60 | 11.20 | 1.29 | 1.29  (-0.44-4.66) |
| ***S100A9*** | 5.59 | 1.71 | 0.44 | 0.17 | 0.43 | 0.22 | 0.19 | 0.40 | 1.14  (-0.16-2.44) |
| ***IRAK3*** | 1.10 | 1.17 | 0.53 | 0.40 | 0.41 | 0.75 | 0.53 | 2.93 | 0.98  (0.40-1.56) |

| **Gene ID** | **Gene expression fold change of 8 individual patients**  **Day 12/Day 0** | | | | | | | | **Mean fold change**  **(95% CI)** |
| --- | --- | --- | --- | --- | --- | --- | --- | --- | --- |
|  | **50-076** | **50-080** | **50-081** | **50-091** | **50-092** | **50-208** | **50-209** | **50-211** |  |
| ***GAS7*** | 5.31 | 0.83 | 0.35 | 0.04 | 0.05 | 0.24 | 0.14 | 0.27 | 0.90  (-0.34-2.15) |
| ***NFKBIA*** | 1.88 | 1.26 | 0.22 | 0.13 | 0.19 | 0.16 | 0.36 | 1.26 | 0.69  (0.21-1.16) |
| ***IL1R2*** | 0.26 | 0.54 | 0.37 | 0.54 | 0.11 | 0.20 | 0.21 | 6.05 | 1.04  (-0.37-2.44) |
| ***S100A9*** | 1.28 | 0.70 | 0.22 | 0.14 | 0.55 | 0.16 | 0.05 | 0.22 | 0.41  (0.13-0.70) |
| ***IRAK3*** | 0.22 | 0.56 | 0.34 | 0.30 | 0.11 | 0.30 | 0.13 | 0.97 | 0.37  (0.17-0.56) |

**Supplementary Table 8.** **Temporal changes in gene expression of melioidosis patients relative to day 0, day 5, and day 12** **(Cont.)**

| **Gene ID** | **Gene expression fold change of 8 individual patients**  **Day 28/Day 0** | | | | | | | | **Mean fold change**  **(95% CI)** |
| --- | --- | --- | --- | --- | --- | --- | --- | --- | --- |
|  | **50-076** | **50-080** | **50-081** | **50-091** | **50-092** | **50-208** | **50-209** | **50-211** |  |
| ***GAS7*** | 7.08 | 1.26 | 0.11 | 0.45 | 0.13 | 0.07 | 0.14 | 0.25 | 1.86  (-0.49-2.86) |
| ***NFKBIA*** | 1.57 | 1.41 | 0.11 | 0.29 | 0.16 | 0.06 | 0.11 | 0.17 | 0.49  (0.52-0.92) |
| ***IL1R2*** | 0.19 | 0.46 | 0.09 | 0.56 | 0.04 | 0.33 | 0.35 | 1.93 | 0.49  (0.07-0.91) |
| ***S100A9*** | 0.56 | 0.91 | 0.15 | 0.14 | 0.35 | 0.11 | 0.04 | 0.10 | 0.30  (0.09-0.50) |
| ***IRAK3*** | 0.19 | 0.59 | 0.19 | 0.72 | 0.08 | 0.16 | 0.17 | 0.36 | 0.31  (0.15-0.47) |

| **Gene ID** | **Gene expression fold change of 8 individual patients**  **Day 12/Day 5** | | | | | | | | **Mean fold change**  **(95% CI)** |
| --- | --- | --- | --- | --- | --- | --- | --- | --- | --- |
|  | **50-076** | **50-080** | **50-081** | **50-091** | **50-092** | **50-208** | **50-209** | **50-211** |  |
| ***GAS7*** | 0.27 | 0.65 | 0.79 | 0.39 | 0.25 | 0.78 | 0.13 | 0.14 | 0.43  (0.23-0.62) |
| ***NFKBIA*** | 0.33 | 0.59 | 0.79 | 0.82 | 0.44 | 0.42 | 0.65 | 0.67 | 0.59  (0.47-0.71) |
| ***IL1R2*** | 0.20 | 0.50 | 0.56 | 0.76 | 0.17 | 0.31 | 0.35 | 0.54 | 0.42  (0.28-0.56) |
| ***S100A9*** | 0.23 | 0.41 | 0.51 | 0.84 | 1.27 | 0.72 | 0.28 | 0.54 | 0.60  (0.37-0.84) |
| ***IRAK3*** | 0.20 | 0.48 | 0.65 | 0.75 | 0.26 | 0.40 | 0.24 | 0.33 | 0.41  (0.28-0.55) |

**Supplementary Table 8. Temporal changes in gene expression of melioidosis patients relative to day 0, day 5, and day 12** **(Cont.)**

| **Gene ID** | **Gene expression fold change of 8 individual patients**  **Day 28/Day 5** | | | | | | | | **Mean fold change**  **(95% CI)** |
| --- | --- | --- | --- | --- | --- | --- | --- | --- | --- |
|  | **50-076** | **50-080** | **50-081** | **50-091** | **50-092** | **50-208** | **50-209** | **50-211** |  |
| ***GAS7*** | 0.36 | 0.98 | 0.24 | 3.99 | 0.61 | 0.25 | 0.13 | 0.13 | 0.84  (-0.07-1.74) |
| ***NFKBIA*** | 0.28 | 0.66 | 0.41 | 1.80 | 0.37 | 0.16 | 0.20 | 0.09 | 0.50  (0.11-0.88) |
| ***IL1R2*** | 0.14 | 0.42 | 0.14 | 0.79 | 0.06 | 0.51 | 0.58 | 0.17 | 0.35  (0.17-0.53) |
| ***S100A9*** | 0.10 | 0.53 | 0.35 | 0.82 | 0.82 | 0.49 | 0.22 | 0.25 | 0.45  (0.26-0.63) |
| ***IRAK3*** | 0.17 | 0.50 | 0.36 | 1.80 | 0.20 | 0.21 | 0.31 | 0.12 | 0.49  (0.07-0.84) |

| **Gene ID** | **Gene expression fold change of 8 individual patients**  **Day 28/Day 12** | | | | | | | | **Mean fold change**  **(95% CI)** |
| --- | --- | --- | --- | --- | --- | --- | --- | --- | --- |
|  | **50-076** | **50-080** | **50-081** | **50-091** | **50-092** | **50-208** | **50-209** | **50-211** |  |
| ***GAS7*** | 1.33 | 1.52 | 0.31 | 10.10 | 2.42 | 0.31 | 1.01 | 0.93 | 2.24  (-0.01-4.49) |
| ***NFKBIA*** | 0.84 | 1.12 | 0.51 | 2.20 | 0.83 | 0.38 | 0.31 | 0.13 | 0.79  (0.34-1.24) |
| ***IL1R2*** | 0.73 | 0.84 | 0.25 | 1.03 | 0.36 | 1.63 | 1.67 | 0.32 | 0.85  (0.46-1.24) |
| ***S100A9*** | 0.44 | 1.30 | 0.68 | 0.97 | 0.64 | 0.68 | 0.79 | 0.46 | 0.75  (0.55-0.94) |
| ***IRAK3*** | 0.88 | 1.05 | 0.56 | 2.38 | 0.74 | 0.53 | 1.27 | 0.37 | 0.97  (0.53-1.42) |

**References**

[1.] Cliff JM, Cho JE, Lee JS, et al. Excessive Cytolytic Responses Predict Tuberculosis Relapse After Apparently Successful Treatment. J Infect Dis. 2016 Feb 1;213(3):485-95.

[2.] Mar AC, Chu CH, Lee HJ, et al. Interleukin-1 Receptor Type 2 Acts with c-Fos to Enhance the Expression of Interleukin-6 and Vascular Endothelial Growth Factor A in Colon Cancer Cells and Induce Angiogenesis. J Biol Chem. 2015 Sep 4;290(36):22212-24.

[3.] Yan LL, Huang YJ, Yi X, et al. Effects of silencing S100A8 and S100A9 with small interfering RNA on the migration of CNE1 nasopharyngeal carcinoma cells. Oncol Lett. 2015 Jun;9(6):2534-2540.

[4.] Zhao Z, Zhong X, Wu T, et al. Identification of a NFKBIA polymorphism associated with lower NFKBIA protein levels and poor survival outcomes in patients with glioblastoma multiforme. Int J Mol Med. 2014 Nov;34(5):1233-40.

[5.] van Uden P, Kenneth NS, Rocha S. Regulation of hypoxia-inducible factor-1alpha by NF-kappaB. Biochem J. 2008 Jun 15;412(3):477-84.

[6.] Bostik P, Dodd GL, Villinger F, et al. Dysregulation of the polo-like kinase pathway in CD4+ T cells is characteristic of pathogenic simian immunodeficiency virus infection. J Virol. 2004 Feb;78(3):1464-72.

[7] Uckun FM, Qazi S, Ma H, et al. CD22DeltaE12 as a molecular target for corrective repair using RNA trans-splicing: anti-leukemic activity of a rationally designed RNA trans-splicing molecule. Integr Biol (Camb). 2015 Feb;7(2):237-49.

[8.] Lu X, Xue L, Sun W, et al. Identification of key pathogenic genes of sepsis based on the Gene Expression Omnibus database. Mol Med Rep. 2018 Feb;17(2):3042-3054.

[9.] Togashi Y, Sakamoto H, Hayashi H, et al. Homozygous deletion of the activin A receptor, type IB gene is associated with an aggressive cancer phenotype in pancreatic cancer. Mol Cancer. 2014 May 27;13:126.
