# Supplementary figures and images for: Blood transcriptomics to characterize key biological pathways and identify biomarkers for predicting mortality in melioidosis
Source: Emerg Microbes Infect. 2021 Jan 17;10(1):8–18. doi: 10.1080/22221751.2020.1858176 (PMC7832033; doi:10.1080/22221751.2020.1858176)

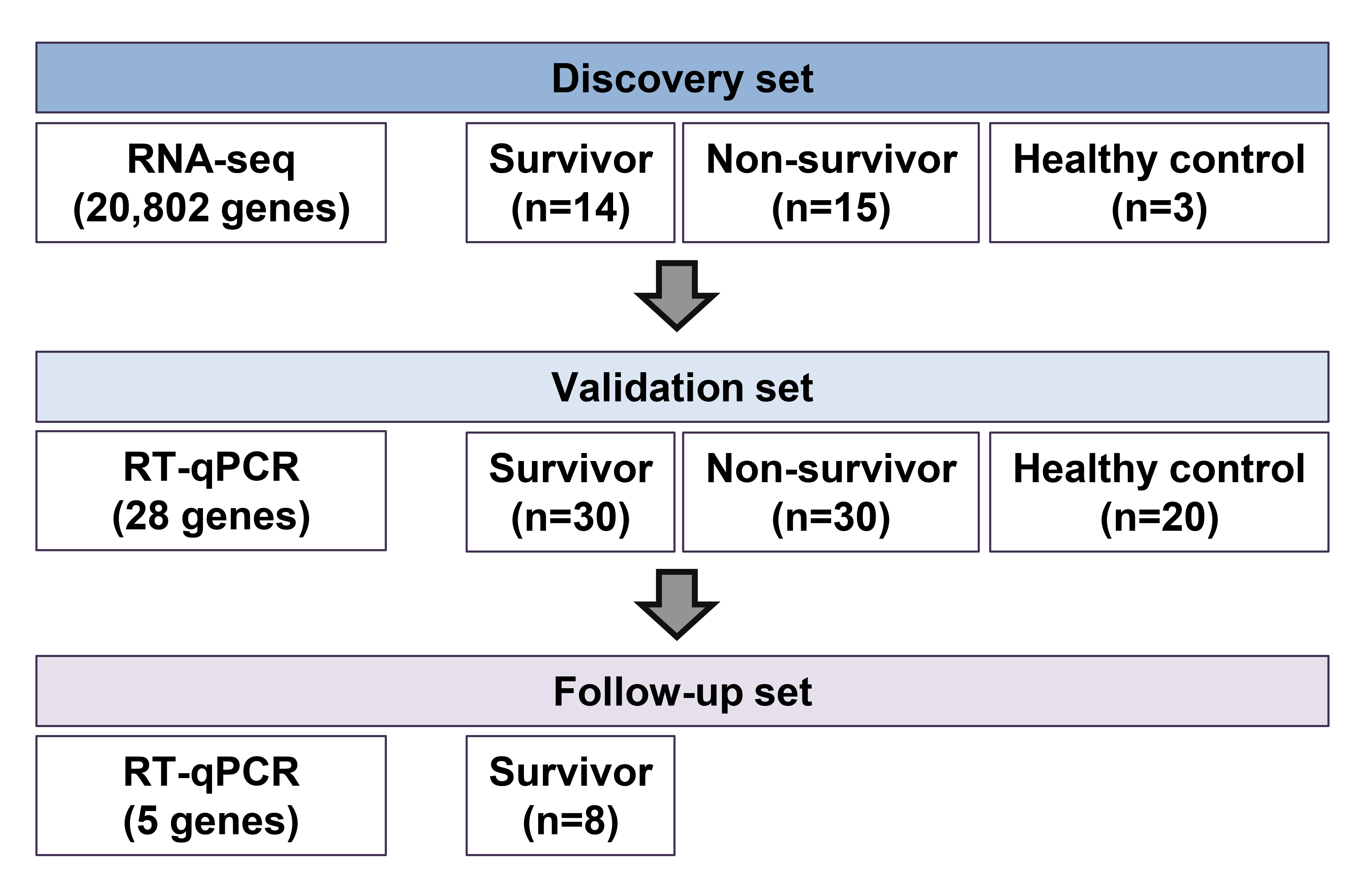

Supplement: Supplementary_Figure_1.tif [file TEMI_A_1858176_SM9796.tif]
